# The cost of ensembling: Is it always worth combining global retail forecasting models?

**DOI:** 10.1371/journal.pone.0336281

**Published:** 2026-09-29

**Authors:** Marco Zanotti

**Affiliations:** Department of Economics, Management and Statistics, University of Milano-Bicocca, Milan, Italy; Universidade Federal do Rio Grande do Sul, BRAZIL

## Abstract

Given the continuous increase in dataset sizes and the complexity of forecasting models, the trade-off between forecast accuracy and computational cost is emerging as an extremely relevant topic, especially in the context of ensemble learning for time series forecasting. To assess it, we evaluated ten base models and eight ensemble configurations across two large-scale retail datasets, considering both point and probabilistic accuracy under varying retraining frequencies. We showed that ensembles consistently improve forecasting performance, particularly in probabilistic settings. However, these gains come at a substantial computational cost, especially for larger, accuracy-driven ensembles. We found that reducing retraining frequency significantly lowers costs, with minimal impact on accuracy, particularly for point forecasts. Moreover, efficiency-driven ensembles offer a strong balance, achieving competitive accuracy with considerably lower costs compared to accuracy-optimized combinations. Most importantly, small ensembles of two or three models are often sufficient to achieve near-optimal results. These findings provide practical guidelines for deploying scalable and cost-efficient forecasting systems, supporting the broader goals of sustainable artificial intelligence in forecasting.

## Introduction

Ensemble learning has become a powerful and widely adopted approach in time series forecasting due to its ability to improve predictive accuracy and robustness by aggregating the outputs of multiple models. The fundamental idea behind ensemble methods is that combining forecasts from diverse models can help balance their individual strengths and weaknesses, thereby reducing the risk of overfitting and mitigating model-specific biases or errors. This approach leverages the principle that while individual models might make different types of errors, their combination can average out these errors. One of the most intriguing and empirically validated findings in the literature is that simple ensemble strategies, such as taking the mean or median of forecasts, often outperform more sophisticated combination methods like stacking (also known as meta-learning) or weighted ensembles. This phenomenon, known as the forecast combination puzzle [1], suggests that in many cases, the added complexity of estimating optimal weights or training a meta-learner does not yield meaningful gains in accuracy and can even lead to overfitting, especially when the number of forecast points is limited. Indeed, the success of simple combinations is partly due to their ability to remain robust in the presence of model misspecification, parameter uncertainty, and changing data dynamics, issues that more complex ensemble methods may struggle to accommodate effectively.

In general, the benefits of ensemble learning are numerous: it improves the forecast accuracy, enhances the model stability across time and datasets, and increases resilience to structural breaks or shifts in the data. Ensembles also provide a practical hedge against model uncertainty, allowing practitioners to rely less on the assumption that any single model is correctly specified. In the context of global forecasting models, ensemble methods become especially valuable. Global models are trained across many time series simultaneously, and while they excel at identifying cross-series patterns, they can also suffer from model instability or biases if the chosen architecture fails to generalize across heterogeneous series. Combining multiple global models, each with different biases, architectures, or feature representations, can help counteract these issues by smoothing out individual model errors and reducing the risk of overfitting to shared but spurious signals across series [2]. Additionally, ensembles serve as a hedge against model selection uncertainty, which is particularly relevant in global settings where the optimal model may vary substantially across groups of series. By pooling predictions, ensembles offer a form of model diversification that improves generalization, making them a powerful and practical tool in modern forecasting systems.

However, ensemble methods are not without drawbacks. One significant limitation is the increased computational cost, especially when combining large numbers of complex models like deep learning architectures or global forecasting models. This cost is magnified when retraining or model tuning is required for each component model at multiple forecast origins. Additionally, from a business perspective, managing and maintaining multiple models adds complexity to the production of forecasts. Moreover, in cases where the component models are very similar or highly correlated, the marginal benefit of ensembling diminishes, potentially offering little improvement over the best individual model [3].

Therefore, one of the major advantages of adopting a global modeling approach, namely scalability, can be offset when ensemble learning is introduced in the forecasting system. Combining several models, even with simple techniques like averaging, inherently increases the computational time, as multiple models must be trained, validated, and maintained, producing substantial computational overhead. In this sense, ensemble learning may weaken one of the core benefits of the global modeling paradigm: reduced training time and resource usage. Moreover, since forecasts are typically generated using cloud computing services with pay-as-you-go pricing models [4], increased computational time and resource consumption directly leads to higher forecasting costs for organizations. These costs may also rise exponentially when frequent retraining strategies are adopted. Indeed, it is a widespread practice to update forecasting models whenever new data becomes available, often driven by the belief that frequent updates enhance the model’s ability to adapt to evolving patterns and improve predictive accuracy. However, when ensembles are used, all the models within the ensembles must be retrained to obtain the final predictions, abruptly increasing the costs of forecasting.

This trade-off between performance and computational time in the context of ensemble learning raises important questions about the balance between accuracy gains and cost efficiency, especially in large-scale forecasting applications. However, despite these challenges, ensemble learning remains one of the most widely used strategies in the forecasting industry, which further amplifies concerns about the long-term sustainability and scalability of forecasting systems. Indeed, from a practical perspective, forecast combinations, coupled with frequent retraining, has also significant environmental costs, since the energy consumption of model training extends beyond direct computational costs, contributing to global energy consumption and carbon emissions [5]. Therefore, understanding the cost of ensembling forecasting models and the effects of retraining on their performance are of paramount importance for advancing more sustainable forecasting practices.

### Research question

We aim to address the question *“Is it always worth combining the predictions of different retail demand forecasting models?.”* Specifically, we focus on the trade-off between accuracy and sustainability, in terms of the cost of producing the forecasts, among different types of ensembles of global forecasting models. This cost is particularly relevant for ensemble models since it becomes exponentially larger with the number of base models used to produce the combined forecasts. To this end, we use ten global models as base learners, ranging from traditional machine learning methods to advanced deep learning architectures. In addition, we simulate several ensemble approaches, all trained and tested on the two most recent and comprehensive retail forecasting datasets: the M5 and VN1 competition datasets. Furthermore, we investigate the effects of retraining on the forecasting performance of ensembles, compared to that of the base models. In particular, we assess whether reducing the frequency of retraining, by avoiding re-estimation of base models as new data becomes available, can effectively enhance the cost-accuracy trade-off in ensemble forecasting. Hence, we explore a wide range of retraining strategies, from continuous retraining to a no-retraining approach, including various intermediate periodic retraining schemes to encompass the most practical and effective scenarios.

### Contributions

Our contribution is fourfold:

- We provide the first comprehensive study of the cost-accuracy trade-off of ensembles of global models, using 10 distinct methods, some of the most relevant real-world retail datasets, and evaluating both point and probabilistic predictions.
- We analyze different ensemble strategies to assess the effects of the ensemble size on forecast accuracy and costs.
- We compare various retraining solutions, such as continuous, periodic, and no retraining, across multiple datasets, to quantify the impact of the retraining frequency on the computational cost of ensemble forecasting.
- We provide practical guidelines for organizations and practitioners on assessing when and how frequently to retrain ensemble forecasting models to obtain an effective balance between predictive accuracy and computational cost.

By tackling these issues, this paper contributes to both forecasting and machine learning communities, offering valuable insights into the trade-offs among accuracy, computational efficiency, and sustainability in the use of an ensemble of global forecasting models.

### Overview

The rest of this paper is organized as follows. After a brief review of related works (Section Related works), in Section Experimental design we describe the design of the experiment used in our study. The datasets and their characteristics are presented in *Datasets*, while the global forecasting models and the ensemble strategies are shown in *Forecasting models*. The concepts related to rolling origin evaluation and retrain scenario are explained in *Evaluation strategy*, while the metrics used to assess the accuracy and cost of models are discussed in *Evaluation metrics*. In Section Results and discussion we show the empirical findings of our study, including forecast accuracy, computing time, and cost analysis of the different scenarios and across the different ensembles. Finally, Section Conclusions contains our summary and conclusions.

## Related works

The cross-learning (global) approach in time series forecasting has witnessed substantial advancements in recent years becoming a central theme in contemporary research. Today, global models are commonly used as benchmarks in empirical studies, underscoring their growing relevance in the field [6]. Additionally, theoretical contributions from [7] and [8] have shown that global models can achieve accuracy comparable to local models with reduced complexity and without relying on assumptions about data similarity. Indeed, global models have proven particularly effective across a variety of forecasting domains, especially in retail forecasting [9], and several methods have been proposed to further boost their performance [10–12]. In particular, ensemble learning is a powerful tool in the forecasters’ hands offering a reliable strategy to enhance accuracy and robustness by combining the strengths of multiple models, not only in the cross-learning context [2]. Relative to point forecasting, many different combination methods have been proposed: linear combinations with optimal weights [13], performance based weighting [14], criteria-based weighting [3], and many different approaches based on stacking, or meta-learning [2]. However, time series forecasting competitions have shown that simple ensemble strategies, like simple mean or median, are extremely accurate, yet efficient [15]. Indeed, simple combination schemes are hard to beat, and the simple arithmetic average of predictions with equal weights remains the most widely used and surprisingly effective combination rule [1].

Most evaluations of global models emphasize point forecast accuracy, likely due to the fact that many machine learning and deep learning algorithms do not natively produce probabilistic outputs [16]. However, in applications such as supply chain management, quantifying uncertainty is essential, whether through prediction intervals, quantiles, or full predictive distributions [17]. Forecast combinations in the context of probabilistic predictions are an active area of research. Different methods have been proposed, from linear pooling, Bayesian model averages, and integral combinations [2]. However, quantile aggregation via simple average has proven to be effective and accurate [18,19].

From the perspective of model retraining, the most comprehensive investigation in the context of global models has been conducted by [20], who found that reducing the retraining frequency of global models can lower forecasting costs without harming accuracy. Other works, such as [21], explored retraining strategies and parameter updates for local exponential smoothing models, while [22] examined retraining in retail demand forecasting using a limited range of models and datasets. Although these studies offer useful insights, direct analysis on the effects of retraining on ensemble models remains unexplored.

The question of whether ensemble methods are consistently worthwhile is relevant both for the forecasting and the broader machine learning community. Indeed, the growing advocacy for sustainable Artificial Intelligence (AI) [5,23] highlights the importance of evaluating the environmental and computational costs of model development and maintenance. Our study directly addresses this gap by systematically evaluating different ensemble strategies’ cost-accuracy trade-off. We also assess how different retraining frequencies affect the forecasting accuracy across the ensemble models, with the aim of testing whether less frequent retraining can be an effective tool to manage this trade-off. Answering these questions, this research provides practical guidelines towards more sustainable forecasting practices.

## Experimental design

This section presents the empirical analysis performed to assess the performance of ensemble models and to determine whether reducing the frequency of retraining can yield forecasting accuracy comparable to that of a baseline scenario, involving continuous retraining, while systematically decreasing the forecasting costs.

### Datasets

For our experiments, we utilized two prominent retail forecasting datasets: the M5 and VN1 competition datasets. The M5 competition, part of the M-competitions series led by Spyros Makridakis and collaborators, was designed to benchmark forecasting methods in a retail demand setting [15]. The M5 dataset [24] is widely recognized and extensively studied, comprising 30,490 daily time series representing unit sales of Walmart products. These sales span three main product categories, Food, Hobbies, and Household, across ten stores in three U.S. states: California, Texas, and Wisconsin. Covering the period from 2011 to 2016, the dataset features highly intermittent time series organized hierarchically, enabling multi-level forecasting (e.g., individual SKUs, categories, stores, and states). It also includes relevant exogenous variables such as prices, promotions, and special events like holidays. The VN1 Forecasting – Accuracy Challenge, launched in October 2024 by Flieber, Syrup Tech, and SupChains, marked the first edition of its kind [25]. This dataset consists of weekly sales data for 15,053 products sold from 2020 to 2024 by various e-vendors, primarily based in the U.S. Unlike the M5 dataset, which contains sales from a single retailer (Walmart) and a limited number of physical stores, the VN1 dataset aggregates sales across 328 warehouses operated by 46 distinct retailers. To the best of our knowledge, we are among the first to benchmark ensemble forecasting models on this dataset. Together, these two datasets represent the most recent and comprehensive publicly available collections of time series related to retail demand, providing a strong foundation for generalizing our findings in the domain of demand forecasting.

Table 1 summarizes the main characteristics of the datasets used in the experiments. The forecasting task is formulated as a global, multi-series, multi-step problem with a single target variable, unit sales, observed at daily (M5) or weekly (VN1) granularity. A single model *F* is estimated jointly on all *S* series of a dataset (Eq 1), using an initial training sample of *n* observations per series. At each forecast origin, inputs are constructed from an input window of *L* observations (which expands by the step size *p* = 1 for each series) of the target (lags and rolling statistics), together with calendar variables, static series identifiers, and, for M5, exogenous event and price variables. The model returns the next *h* values of the same target variable at the same granularity: 28 days for M5 and 13 weeks for VN1. Machine learning models generate the *h* values recursively, whereas deep learning models produce them directly in a single multi-output step. We deliberately retained each framework’s native strategy rather than forcing a common one.

**Table 1:** Characteristics of the datasets.

	M5	VN1
Number of series (*S*)	30,490	15,053
Time step	1 day	1 week
Seasonal period (*m*)	7	52
Target variable	unit sales	unit sales
Training length (*n*)	1,460 (4 years)	157 (3 years)
Input window (*L*)	44,515,400	2,363,321
Step size (*p*)	1	1
Test length (*T*)	364 (1 year)	52 (1 year)
Horizon (*h*)	28 (4 weeks)	13 (1 quarter)
Forecast origins	336	39

In both datasets, we concentrated on the most disaggregated level (SKU × Store), because the potential gains from reduced retraining are greatest at lower aggregation levels. To ensure a consistent evaluation strategy (see Section Evaluation strategy), we filtered the series: daily SKUs retained at least two years of data (more than 730 observations), while weekly SKUs required a minimum of three years (more than 157 observations). No missing data were present in the final datasets.

### Forecasting models

In this study, we focused exclusively on global forecasting methods, as we used only this category of models as base learners for our ensembles The analysis of local models is out of the scope of our study. A global forecasting model can be defined as:


$$\begin{array}{c} Y_{i}^{h}=F(𝒴,\Theta )\text{,} \end{array}$$ (1)

where forecasts for a horizon *h* for each individual time series $Y_{i}$ are generated using a single model *F* trained on the entire set of time series in a dataset 𝒴. Hence, using the global modeling paradigm, it is possible to leverage cross-learning to allow the model to learn shared patterns across all time series. Indeed, the model parameters Θ are shared across all series.

To ensure a comprehensive evaluation of the ensemble models, we included both traditional machine learning models and state-of-the-art deep learning techniques as base learners, chosen for their proven effectiveness in time series forecasting and their methodological diversity. Additionally, given the importance of evaluating forecasting models against established benchmarks in time series analysis, we adopted two well-known global models, Linear (Pooled) Regression and Multi-Layer Perceptron, as reference methods for comparison throughout our experiments [26]. Table 2 provides all the time series forecasting models used.

**Table 2:** Global forecasting models used as base models.

Linear Regression, LR [26]
Random Forest, RF [27]
Extreme Gradient Boosting, XGBoost [28]
Light Gradient Boosting Machines, LGBM [29]
Categorical Boosting, CatBoost [30]
Multi-Layer Perceptron, MLP [31]
Long-Short Term Memory, LSTM [32]
Temporal Convolution Network, TCN [33]
Neural Basis Expansion, NBEATS [34]
Neural Hierarchical Interpolation, NHITS [35]

As in [20], we implemented simplified feature engineering pipelines inspired by the top-performing solutions of the M5 and VN1 competitions. Our feature set included standard time series features such as lags, rolling and expanding means, as well as calendar-related variables like year, month, week, and day of the week. We also integrated static metadata, such as store, product, category, and location identifiers, based on the dataset’s characteristics. For the M5 dataset, we further included external variables like special events. Hyperparameters were chosen based on configurations from leading competition entries when available. Otherwise, we used the default values recommended by the respective software libraries (see Supporting information). No hyperparameter optimization procedure was adopted.

### Ensemble learning

Ensemble learning aggregates the predictions of multiple base models to enhance forecast accuracy and reliability, especially when different models capture diverse and complementary patterns in the data. This approach is particularly beneficial in global forecasting, where the use of a single model can lead to instability or overfitting due to the heterogeneous nature of the time series being modeled [2]. To address these challenges, forecast combinations help by balancing out the individual biases and variances of the base models. In a general formulation, an ensemble forecasting model combines the outputs of multiple models. Using the same notation introduced before, where 𝒴 is the set of all time series and $\Theta^{\left(j\right)}$ denotes the parameters of the *j*-th base model *F*^(*j*)^, the ensemble prediction for series $Y_{i}$ at horizon *h* can be defined as:


$$\begin{array}{c} Y_{i}^{h}=G\left(F^{\left(1\right)}(𝒴,\Theta^{\left(1\right)}),F^{\left(2\right)}(𝒴,\Theta^{\left(2\right)}),...,F^{\left(J\right)}(𝒴,\Theta^{\left(J\right)})\right)\text{.} \end{array}$$ (2)

Here, *G* is the ensemble function (e.g., mean, median, weighted average) that combines the forecasts of *J* base models.

In our study, we adopted an ensemble approach that combines the models’ predictions using a simple average. The simple average is a widely used method in forecasting to create forecast combinations due to its simplicity, proven effectiveness in improving prediction robustness, and is often more accurate than theoretically optimal combinations [1]. Therefore, being *G* the simple mean, the ensemble forecast for series $Y_{i}$ at horizon *h* becomes:


$$\begin{array}{c} Y_{i}^{h}=\frac{1}{J}\sum_{j=1}^{J}F^{\left(j\right)}(𝒴,\Theta^{\left(j\right)})\text{.} \end{array}$$ (3)

Furthermore, since we are not only interested in point forecast accuracy, in the context of probabilistic forecasting, ensemble methods can also be applied to combine predictive distributions. A common and straightforward approach is to average the predicted quantiles across base models [2]. That is, for each desired quantile level (e.g., 0.1, 0.5, 0.9), the ensemble forecast simply takes the mean of the corresponding quantiles predicted by the individual models. Despite its simplicity, this method is often effective and overall seems to be preferred compared to other combination techniques [18]. Moreover, equally weighting quantiles through a simple average yields robust and improved forecast results because the error in estimating optimal weights usually exacerbates the ensemble predictions [19]. Lastly, simple combination methods have the lowest computational time possible, allowing us to effectively study the associated costs of ensembling without any loss of generality. Indeed, simple combinations, such as the mean or median, require only basic arithmetic operations on the forecast outputs of the base models, avoiding the need for model training or optimization.

It is important to note that alternative ensemble strategies exist beyond simple averaging, including weighted combinations, dynamically adjusted weights, and meta-learning approaches such as stacking [2]. These methods can potentially improve predictive accuracy by assigning higher importance to more reliable models or adapting the combination scheme to different regions of the feature space. However, such approaches typically introduce additional computational overhead due to the need for weight estimation, validation procedures, or secondary learning models. In the context of this study, where the focus is on evaluating the trade-off between forecast accuracy and computational cost under repeated retraining settings, we restrict our analysis to simple averaging ensembles. This choice provides a strong, widely used baseline while ensuring minimal additional complexity, allowing us to isolate and study the cost implications of ensembling in a transparent manner.

In our study, we simulate various ensemble strategies to assess, a posteriori, how forecast performance over the full test window would have changed if forecasting combinations had been used in place of the individual base models. Hence, we employed simple mean ensembles to combine the forecasts of multiple pools of global models, following two distinct selection criteria. The first strategy, which we refer to as *Ensemble Accuracy* (*ENSACC*), combines the base models that demonstrated the highest individual point forecast accuracy (measured by Eq 4). This approach aims to leverage the strengths of top-performing models, under the assumption that their combined output will retain strong predictive performance while potentially offsetting individual weaknesses. The second strategy, *Ensemble Time* (*ENSTIME*), focuses instead on computational efficiency by combining the models with the lowest training and inference times. This ensemble reflects a pragmatic choice for real-world forecasting systems where computational cost is a critical constraint, such as in large-scale retail applications. For both strategies, we constructed ensembles of increasing size, combining the top 2, 3, 4, and 5 models according to each criterion (see Table 3). This idea of only using a subset of the base learners in ensembling is essentially a variant of model pooling, as defined by [36]. This tiered design allows us to assess how forecast accuracy and computational efficiency evolve as additional models are added to the ensemble. Limiting the maximum ensemble size to five models (out of eight or ten, depending on the dataset) reflects a balance between potential accuracy gains and the diminishing returns or increased complexity often observed with larger ensembles [2]. This systematic approach enables a thorough evaluation of the trade-off between accuracy and computational cost in ensemble-based global forecasting.

**Table 3:** Composition of ENSACC and ENSTIME ensembles.

Dataset	Ensemble	Base Models Included
M5	**ENSACC (Accuracy-driven combinations)**	
	Ens2A	XGBoost, LGBM
	Ens3A	XGBoost, LGBM, LR
	Ens4A	XGBoost, LGBM, LR, NBEATSx
	Ens5A	XGBoost, LGBM, LR, NBEATSx, MLP
	**ENSTIME (Time-efficient combinations)**	
	Ens2T	CatBoost, LR
	Ens3T	CatBoost, LR, XGBoost
	Ens4T	CatBoost, LR, XGBoost, MLP
	Ens5T	CatBoost, LR, XGBoost, MLP, NBEATSx
VN1	**ENSACC (Accuracy-driven combinations)**	
	Ens2A	MLP, NBEATSx
	Ens3A	MLP, NBEATSx, NHITS
	Ens4A	MLP, NBEATSx, NHITS, RF
	Ens5A	MLP, NBEATSx, NHITS, RF, XGBoost
	**ENSTIME (Time-efficient combinations)**	
	Ens2T	LR, XGBoost
	Ens3T	LR, XGBoost, CatBoost
	Ens4T	LR, XGBoost, CatBoost, MLP
	Ens5T	LR, XGBoost, CatBoost, MLP, TCN

All models were implemented in Python using the open-source Nixtla’s framework [37]. Specifically, the *mlforecast* library was used to train the machine learning models, while *neuralforecast* was employed for efficiently training the deep learning models.

### Evaluation strategy

Following [20], we evaluated multiple retraining scenarios, or retrain windows. A retrain scenario *r* denotes a positive integer indicating how often the model is updated, or retrained. Specifically, *r* defines the number of new observations that must be collected before retraining occurs. These scenarios are tailored to the frequency of each dataset, which in turn determines the forecast horizon and business review cycles. Table 4 summarizes the selected retraining sets.

**Table 4:** Retraining sets. The setup is based on the respective time series frequency.

Dataset	Retraining set
M5	{7, 14, 21, 30, 60, 90, 120, 150, 180, 364}
VN1	{1, 2, 3, 4, 6, 8, 10, 13, 26, 52}

For example, *r* = 7 for daily data implies weekly retraining. Each list contains ten scenarios to ensure a broad yet computationally feasible exploration. The scenario with *r* = 1, known as continuous retraining, is the most computationally intensive and typically the most accurate, as the model always uses the most recent data. Hence, we adopt it as our benchmark for both accuracy and cost. However, for daily data, we consider *r* = 7 (weekly retraining) as the practical benchmark, since daily updates are uncommon in real-world settings. Conversely, the no-retraining scenario *r* = *T*, where *T* is the length of the test set, fits the model only once, and uses it for the entire forecasting horizon, minimizing computational load but likely yielding the lowest accuracy. All intermediate values 1 < *r* < *T* represent periodic retraining strategies, where both forecast accuracy and computational cost are expected to decrease as *r* increases.

In time series forecasting out-of-sample evaluation is crucial [38], and rolling origin evaluation is widely regarded as the most reliable method because it systematically simulates repeated forecasting cycles and averages performance over multiple iterations [39]. In our study, we adopt an expanding window setup with a one-year test set, business-relevant forecast horizons, and a step size of one to ensure a realistic and comprehensive evaluation. Fig 1 shows the whole evaluation process.

**Fig 1:**
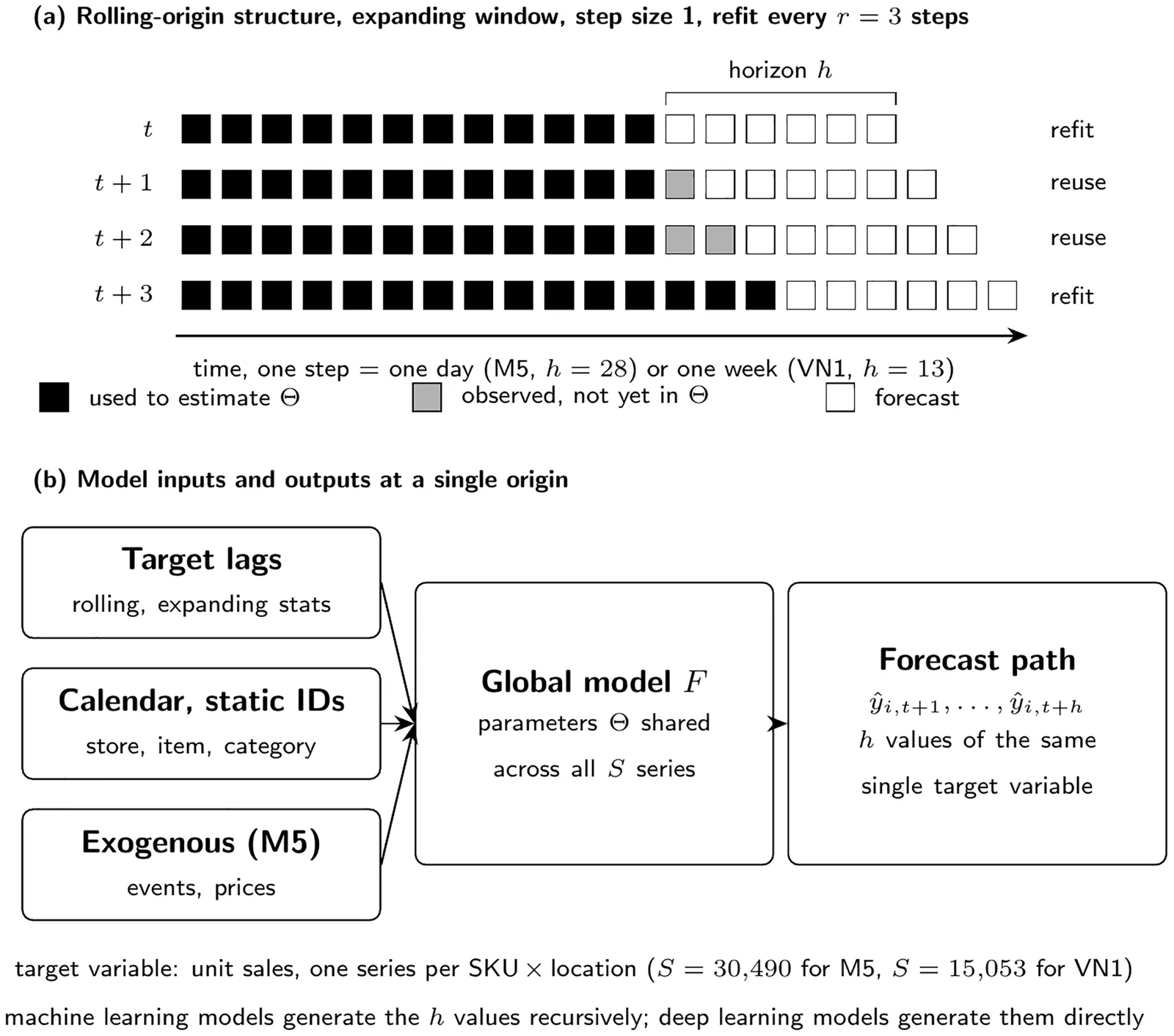
Rolling-origin structure and input–output mapping of the global forecasting evaluation strategy.

### Evaluation metrics

Evaluating the accuracy of point forecasts in time series analysis remains a debated topic. Although numerous metrics exist to assess model performance, there is no clear consensus in the literature on which metric is superior [40]. Given that our focus is on SKU-level demand forecasting, where data is typically intermittent, error metrics based on absolute or percentage deviations are suboptimal, as they emphasize the median rather than the full distribution [41]. Furthermore, due to scale variations across series, employing a scale-independent metric is essential. To address these concerns, we adopted the Root Mean Squared Scaled Error (RMSSE), introduced by [42], as our primary point forecast accuracy measure. RMSSE is calculated as:


$$\begin{array}{c} \text{RMSSE}=\sqrt{\frac{\frac{1}{h}\sum_{t=n+1}^{n+h}(y_{t}-{\hat{y}}_{t}{)}^{2}}{\frac{1}{n-s}\sum_{t=s+1}^{n}(y_{t}-y_{t-s}{)}^{2}}}\text{.} \end{array}$$ (4)

This metric compares the mean squared error of a forecast to that of a seasonal naïve benchmark, providing a relative performance measure. It was the official evaluation metric used in the M5 competition (with *s* = 1, hence using the naive model as a benchmark) [15]. Lower RMSSE values indicate more accurate forecasts.

Beyond point accuracy, we also assessed the probabilistic quality of forecasts across different retraining scenarios. To evaluate the quality of the estimated quantiles, we used a scaled version of the Quantile Loss (SQL), also known as Pinball Loss, and its average form, the scaled Multi-Quantile Loss (SMQL):


$$\begin{array}{c} \text{SQL}(\alpha )=\frac{\frac{1}{h}\sum_{t=n+1}^{n+h}(\alpha \cdot (y_{t}-{\hat{q}}_{t}^{\left(\alpha \right)})\cdot {𝕀}_{{\hat{q}}_{t}^{\left(\alpha \right)}\leq y_{t}}+(1-\alpha )\cdot ({\hat{q}}_{t}^{\left(\alpha \right)}-y_{t})\cdot {𝕀}_{{\hat{q}}_{t}^{\left(\alpha \right)}>y_{t}})}{\frac{1}{n-s}\sum_{t=s+1}^{n}|y_{t}-y_{t-s}|}\text{,} \end{array}$$ (5)

$$\begin{array}{c} \text{SMQL}=\frac{1}{\left|𝒬\right|}\sum_{\alpha \in 𝒬}\text{SQL}(\alpha )\text{,} \end{array}$$ (6)

where 𝒬 is the set of possible quantile levels α, and ${\hat{q}}_{t}^{\left(\alpha \right)}$ is the quantile forecast for level α. As a proper scoring rule, QL enables a rigorous evaluation of probabilistic forecasts [43]. The scaling factor is the in-sample, one-step-ahead mean absolute error of the seasonal Naive model. The scaled MQL, particularly in its weighted form and with *s* = 1, was the principal metric of the M5 Uncertainty competition [16]. For daily data we used *s* = 7, because *s* = 1 is arguably an irrelevant benchmark in daily retail data, which typically exhibits strong day-of-week seasonality [17]. Instead, we set *s* = 1 for weekly data.

We adopted Conformal Inference as a general framework for producing quantile forecasts (See Supporting information), considering a total of 14 different quantiles of probability levels α∈ {0.005, 0.025, 0.050, 0.100, 0.150, 0.200, 0.250, 0.750, 0.800, 0.850, 0.900, 0.950, 0.975, 0.995}. Therefore, we studied the 50% 60%, 70%, 80%, 90%, 95%, and 99% symmetric prediction intervals. The lower intervals (e.g., 50%, and 60%) describe the forecast center, while the upper intervals (90% and above) are crucial for assessing tail risks, key to managing safety stock in retail settings [44]. These quantiles offer a well-rounded view of forecast uncertainty.

In addition to accuracy, we explicitly evaluated the computational cost of generating forecasts by measuring Computing Time (CT), defined as the number of seconds needed to train a model and produce *h* step-ahead forecasts [20]. CT directly reflects forecasting costs in real-world applications, especially when using cloud-based infrastructure with pay-as-you-go pricing [21]. Thus, CT was recorded for each model across all retraining scenarios, with lower CT values indicating more efficient forecasting.

To facilitate consistent comparisons, results for all evaluation metrics were normalized relative to the baseline (continuous) retraining scenario for each dataset frequency. To statistically assess performance differences across scenarios, we applied the Friedman-Nemenyi test for multiple comparisons [45]. The whole pipeline is depicted in the Supplementary material.

All experiments were conducted using a Microsoft Azure NC6s_v3 cloud instance running Ubuntu 24, equipped with 1 GPU, 6 CPU cores, and 112 GB of memory.

## Results and discussion

This section presents and interprets the empirical findings of our study, highlighting the interplay between accuracy, probabilistic performance, computing time, and total cost across different retraining strategies and ensemble configurations. We draw insights from both the M5 and VN1 datasets to identify consistent patterns and dataset-specific nuances.

Table 5 summarizes the performance and computational time of all forecasting methods evaluated in this study, across both the M5 and VN1 datasets. Minimum RMSSE and SMQL values per column are highlighted in bold. The baseline scenarios are *r* = 7 for the M5 dataset and *r* = 1 for the VN1 dataset. The models are grouped into four categories: Machine Learning (ML), Deep Learning (DL), ENSACC (ensembles optimized for accuracy), and ENSTIME (ensembles optimized for computational efficiency). For each method, the table reports three key metrics per dataset: RMSSE (Root Mean Squared Scaled Error) to assess point forecast accuracy, SMQL (Scaled Multi-Quantile Loss) to evaluate probabilistic forecast performance, and CT (Computing Time, in seconds) to reflect the computational time under a cloud-based setting.

**Table 5:** Performance and computational time across datasets.

Type	Method	M5			VN1		
		RMSSE	SMQL	CT	RMSSE	SMQL	CT
Base models	LR	0.760	0.269	11373	6.535	4.005	**236**
	RF	–	–	–	1.871	1.220	24862
	XGBoost	**0.739**	0.246	15417	1.898	1.503	530
	LGBM	0.755	0.247	44429	3.540	2.318	7824
	CatBoost	0.926	0.280	**10424**	5.355	3.889	805
	MLP	0.804	0.264	17584	1.554	**0.806**	962
	LSTM	–	–	–	1.960	0.892	1284
	TCN	0.847	0.271	33364	1.960	0.891	1127
	NBEATSx	0.799	0.263	21226	1.704	0.991	1244
	NHITS	0.812	0.267	21969	1.705	0.992	1251
Ensembles	Ens2A	0.741	0.244	59846	**1.478**	0.826	2205
	Ens3A	0.741	0.248	71219	1.523	0.867	3456
	Ens4A	0.743	**0.241**	92445	1.530	0.926	28318
	Ens5A	0.748	0.242	110029	1.550	1.013	28849
	Ens2T	0.798	0.257	21797	5.694	4.388	766
	Ens3T	0.762	0.249	37214	5.286	3.972	1572
	Ens4T	0.751	0.242	54797	4.447	3.420	2533
	Ens5T	0.750	0.243	76024	3.664	2.832	3660

Overall, the models evaluated achieved better absolute performance on the M5 dataset than on VN1. The overall results are derived from the baseline scenario, that is *r* = 7 for M5 and *r* = 1 for VN1, as this setting is regarded as standard in both theoretical and practical applications. Several factors may explain this difference, including the larger dataset size, the higher frequency of the time series in M5, the availability of rich external regressors (e.g., promotions, special events), and the presence of well-established benchmark hyperparameter settings, many of which were not available for VN1 during model training.

Analyzing the point forecasting accuracy, the RMSSE confirms the effectiveness of ensemble methods in both datasets. In general, ENSACC ensembles (those optimized for accuracy) consistently outperform individual base models. The accuracy improvements, however, show diminishing returns beyond three or four models, aligning with the well-documented phenomenon in ensemble literature where the marginal gain from adding more models decreases. Notably, while ENSACC ensembles dominate in accuracy, ENSTIME ensembles (optimized for computational efficiency) still provide competitive results in the M5 dataset, demonstrating that even time-efficient combinations can retain good predictive power. Interestingly, ENSTIME ensembles show increasing returns in accuracy as more models are added: their performance consistently improves with each additional component. Indeed, in contrast to ENSACC ensembles, where combining just two models is often sufficient to achieve optimal performance, ENSTIME combinations benefit from larger ensemble sizes, with forecast accuracy continuing to improve as more low-cost models are included.

A similar pattern emerges for probabilistic accuracy evaluated using Scaled Multi-Quantile Loss (SMQL). Compared to base models, ensembles achieve superior uncertainty quantification, benefiting from the diversity in quantile predictions among different models. Simple averaging across quantiles proves to be a highly effective strategy. Also, in this case, ENSACC forecast combinations exhibit diminishing returns in accuracy as more models are added. On the contrary, increasing the number of models in the ensemble based on computational efficiency leads to consistently improved probabilistic forecasting performance, which may even outperform the ENSACC combinations. ENSTIME ensembles achieve similar probabilistic forecasting performance on the M5 dataset compared to all accuracy-based combinations, while their performance is worse on the VN1 dataset. In contrast, for ENSACC ensembles, combining more than two base models does not yield additional improvements, confirming once again that a small, high-performing subset is sufficient in that case.

Unsurprisingly, ensemble models require significantly more computing time than individual models, with algorithms like LGBM and RF contributing disproportionately to this overhead. ENSACC ensembles are especially time-intensive, as they often include the most computationally demanding models. In contrast, ENSTIME ensembles maintain a more manageable runtime profile by design, though they still incur a higher computational cost than their individual components due to the cumulative effect of combining multiple models.

Fig 2 presents the point forecast accuracy of each ensemble model across various retraining scenarios for both the M5 and VN1 datasets. To facilitate comparison, the results are expressed in relative terms with respect to the baseline retraining scenarios (*r* = 7 for M5 and *r* = 1 for VN1).

**Fig 2:**
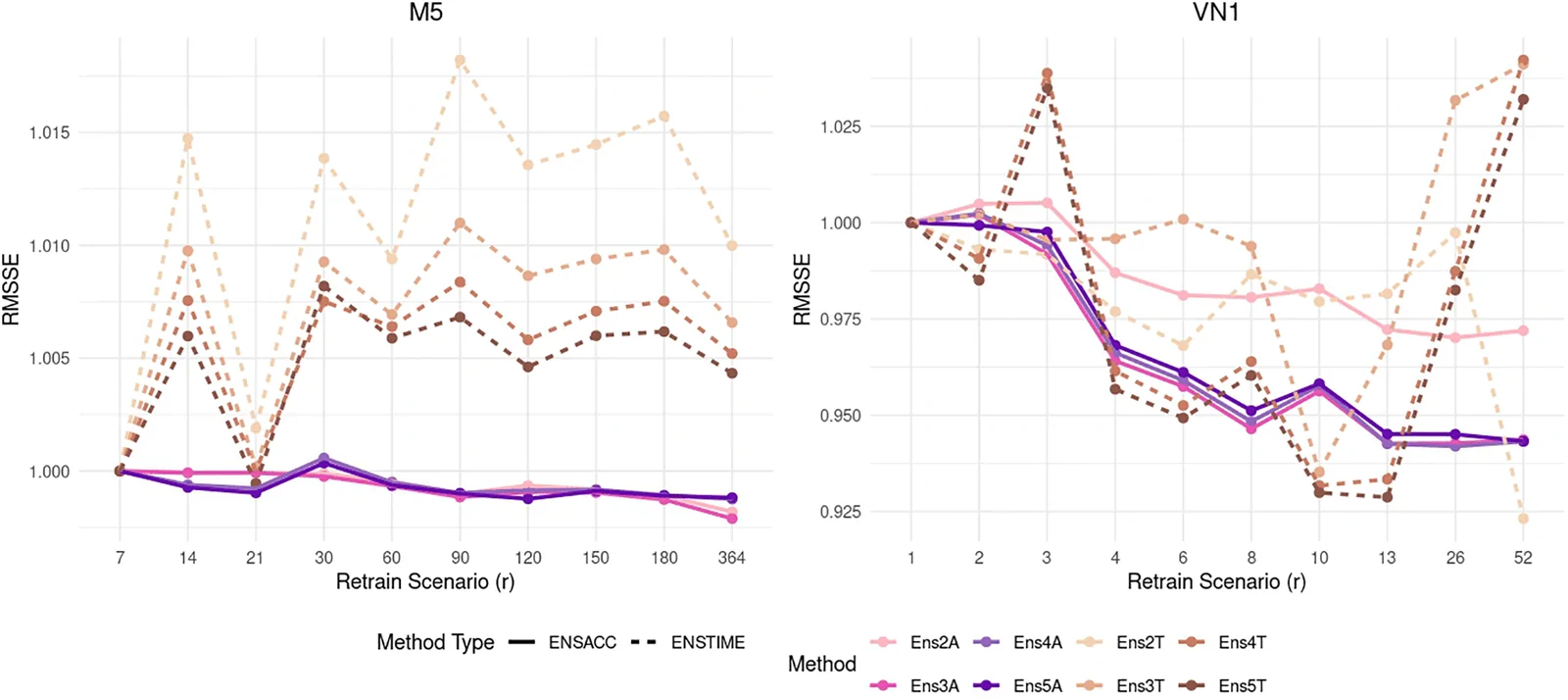
RMSSE results for each ensemble and retrain scenario combination in relative terms with respect to the baseline scenario, *r* = 7 for the M5 dataset and *r* = 1 for the VN1 dataset.

The RMSSE trajectories of the ENSACC ensembles exhibit remarkable stability across retraining periods. In the M5 dataset, their accuracy remains virtually unchanged regardless of retraining frequency, while in the VN1 dataset, performance even improves in some scenarios. For most ensemble models, particularly under low-frequency retraining (i.e., periodic updates), performance is nearly indistinguishable from the baseline. Although slight accuracy degradation is observed for the ENSTIME ensembles under less frequent retraining, it remains modest, below 2% in M5 and under 3% in VN1, even in the no-retraining condition. These findings build upon and extend the results of [20], suggesting that reducing retraining frequency does not significantly harm the point forecast accuracy of global ensemble models. This robustness likely stems from the relative stability of the datasets, which do not exhibit substantial structural changes or concept drift. Under such conditions, base models continue to capture underlying demand patterns effectively over time, and ensemble combinations further enhance stability as retraining becomes less frequent. For this reason, these results should be interpreted under conditions of moderate and gradual data evolution, as observed in the considered datasets (more frequent retraining may still be necessary to maintain optimal performance).

Similarly, Fig 3 illustrates the relative accuracy of the ensemble models in a probabilistic forecasting context, as measured by the Scaled Multi-Quantile Loss (SMQL).

**Fig 3:**
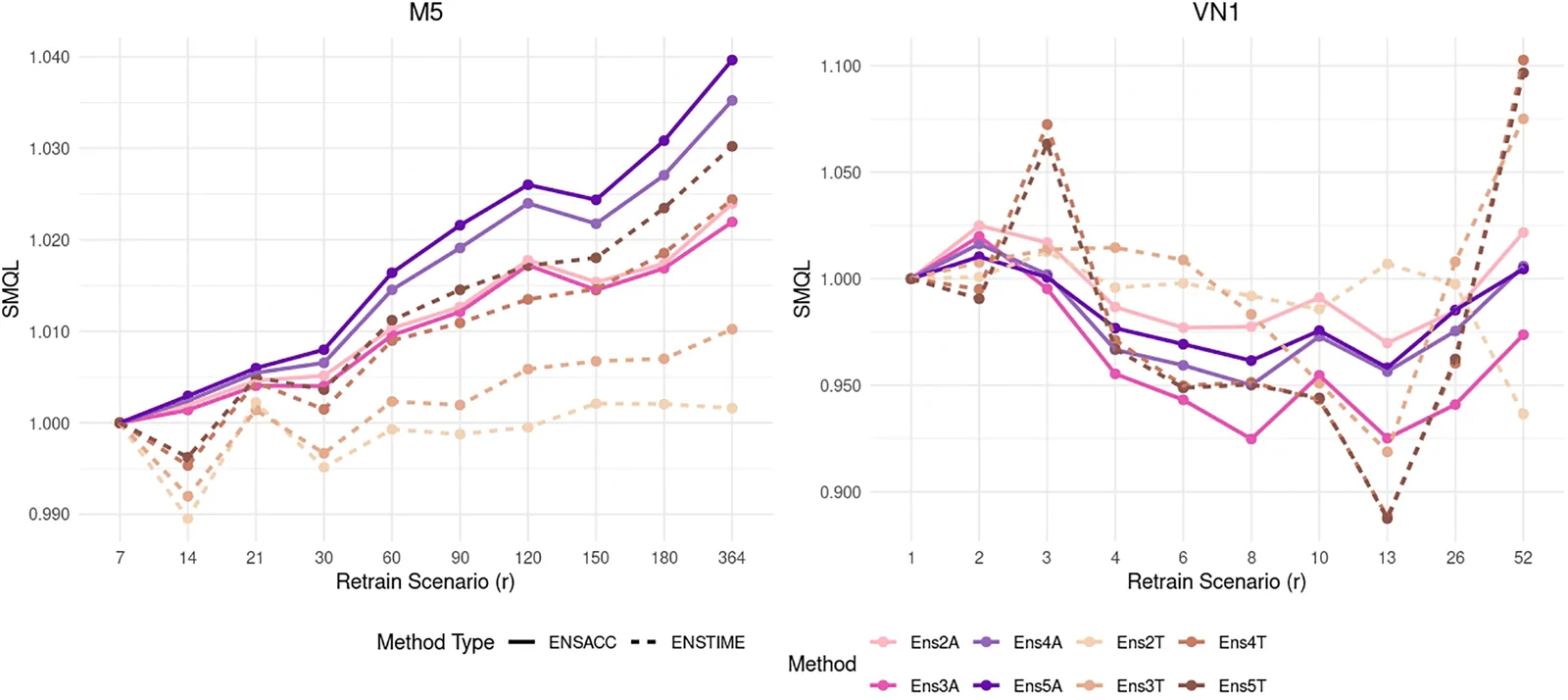
SMQL results for each ensemble and retrain scenario combination in relative terms with respect to the baseline scenario, *r* = 7 for the M5 dataset and *r* = 1 for the VN1 dataset.

For the M5 dataset, we observe a clear trend where probabilistic accuracy decreases as retraining becomes less frequent, indicating that regular updates are beneficial for maintaining high-quality uncertainty estimates. This pattern holds across the different ensemble strategies. For ENSACC ensembles, the decline in accuracy is minimal for frequent retraining scenarios and becomes slightly more noticeable at higher levels of retraining, though it remains within 4 percentage points. ENSTIME combinations, instead, show even higher performance, in particular in the case of the smallest ensembles.

In contrast, the VN1 dataset reveals a different behavior. Here, the relationship between retraining frequency and probabilistic accuracy follows a near-convex pattern. Performance initially improves with less frequent retraining, peaking around *r* = 8, and begins to decline thereafter. This suggests that very frequent updates may not be necessary for probabilistic performance in stable, lower-frequency datasets like VN1. Interestingly, the rate of performance deterioration with increased retraining window is comparable across both ensemble strategies in VN1, indicating that the marginal impact of retraining frequency is relatively uniform across the proposed ensemble techniques. Also these results on the probabilistic performance of ensembles are comparable to those obtained by [20] on the base models.

The Friedman-Nemenyi tests, used to compare the accuracy produced by the same model over different retraining scenarios, confirm the above results (See Supplementary information). Specifically, in the evaluation of both point forecast accuracy and probabilistic forecast accuracy, some level of periodic retraining is at least as good as the continuous retraining scenario.

Fig 4 illustrates the relative computational time (CT) across different retraining scenarios for both the M5 and VN1 datasets. As anticipated, CT decreases sharply as the retraining interval increases, with the reduction following an approximately exponential pattern. This effect is more pronounced in the VN1 dataset, likely due to its smaller size compared to M5. Specifically, moving from the baseline retraining scenario to the first periodic setting (*r* = 14) results in a roughly 33% reduction in computing time for M5, while for VN1, CT is nearly halved.

**Fig 4:**
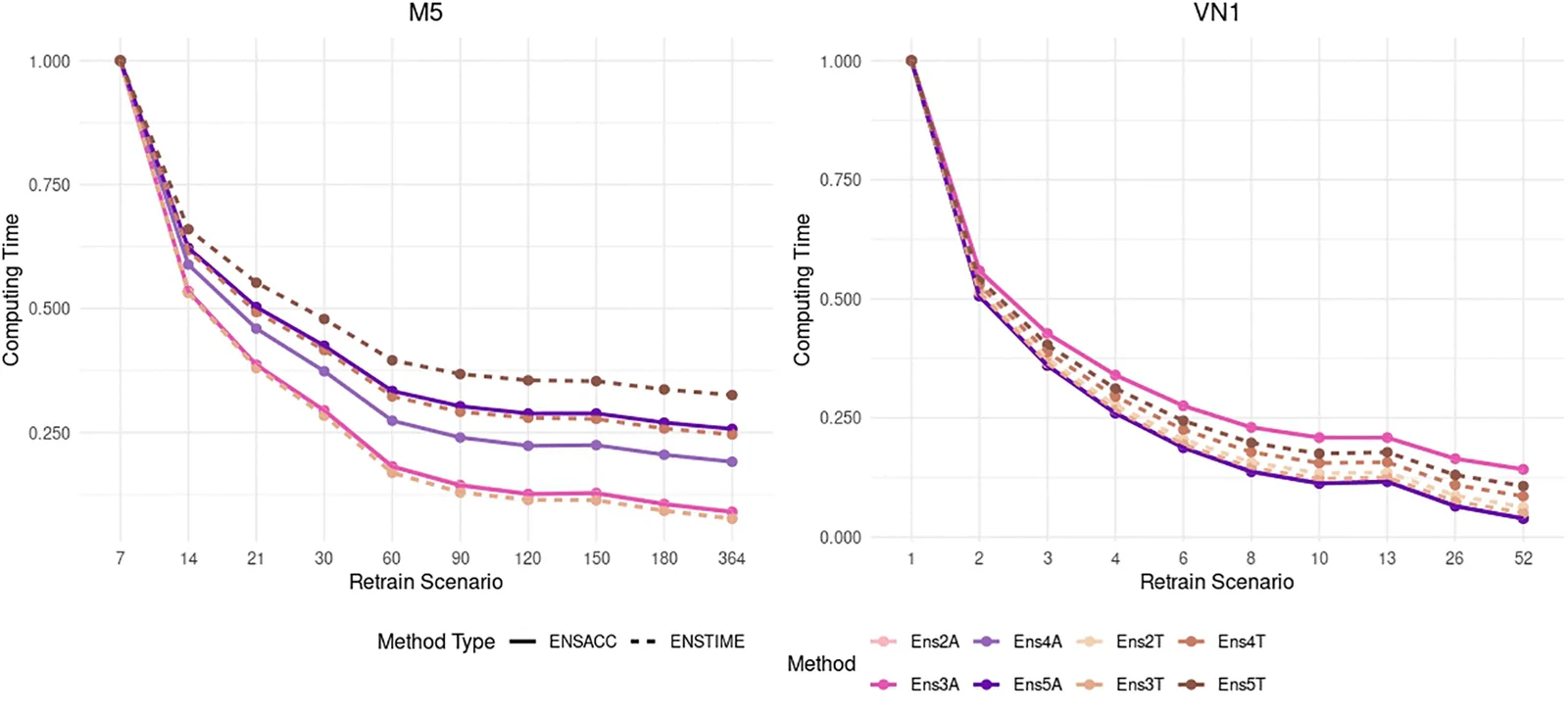
CT results for each ensemble and retrain scenario combination in relative terms with respect to the baseline scenario, *r* = 7 for the M5 dataset and *r* = 1 for the VN1 dataset.

As dataset size increases, the marginal gains in CT from reducing retraining frequency appear to depend more on the number of models in the ensemble (ensemble depth) than on the ensemble type (ENSACC vs. ENSTIME). Simpler ensembles composed of only 2 or 3 models benefit most from reduced retraining, exhibiting steeper declines in CT. Moreover, ENSACC and ENSTIME ensembles show very similar CT profiles when matched by depth, suggesting that computational efficiency in this context is more a function of ensemble size than selection strategy. This distinction is less pronounced in the VN1 dataset, given its smaller scale and shorter time series. These findings have direct implications for forecasting cost management, emphasizing the importance of considering retraining frequency and ensemble size when selecting models for large-scale operational use.

Overall, the evidence from Figs 2, 3, and 4, along with the statistical significance tests, indicates that reducing the retraining frequency of ensemble models does not negatively impact their predictive accuracy. At the same time, it lowers the computational time required to generate forecasts. However, effectively leveraging this time reduction depends on carefully considering the ensemble size. As more models are included in the ensemble, the relative benefit of periodic retraining diminishes compared to using only a single model as in [20]. Consequently, both the retraining frequency and the ensemble depth play a critical role in managing computational efficiency, and ultimately the cost, of ensemble-based forecasting systems.

As previously noted, computational time can be directly translated into monetary cost for organizations. In line with [4,20,46], we adopted standard pricing assumptions for cloud computing services to estimate both the overall cost of forecasting and the specific costs associated with each retraining scenario for the base global models and each ensemble. The costs have been normalized by the number of SKUs in each dataset to allow comparisons. Figs 5, 6, and 7 illustrate the estimated forecasting costs for a large-scale retailer, assuming a computing service rate of $3.5 per hour, with 200,000 unique SKUs and 5,000 stores. It is important to note that the cost analysis is based on a simplified and controlled experimental setup, which assumes a fixed hardware configuration and a constant pricing scheme. This design choice ensures comparability across models and retraining scenarios and follows established practice in the forecasting literature on retraining strategies, including [21], which similarly adopts fixed computational assumptions to study cost-related effects. While real-world forecasting systems may exploit heterogeneous infrastructure, parallel execution, and dynamic pricing schemes, such factors primarily affect the absolute magnitude of computational costs rather than the relative differences between methods. Therefore, the reported results should be interpreted in terms of relative cost–accuracy trade-offs rather than precise operational cost estimates. In particular, although more complex ensemble strategies may increase computational requirements under the adopted setup, the qualitative comparison between single models and ensembles remains informative under consistent evaluation conditions.

**Fig 5:**
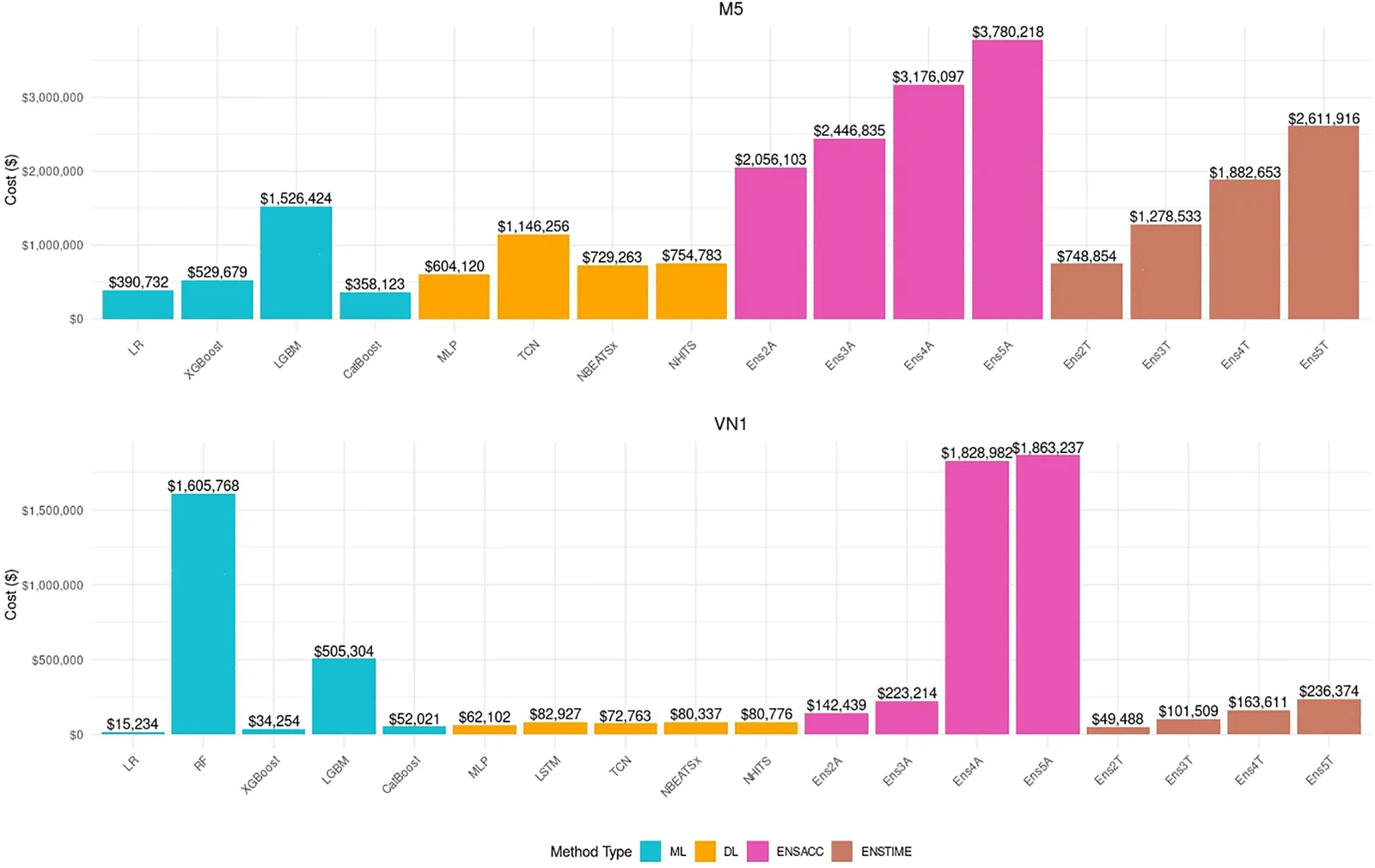
Cost analysis. Overall cost results for the M5 and VN1 datasets.

**Fig 6:**
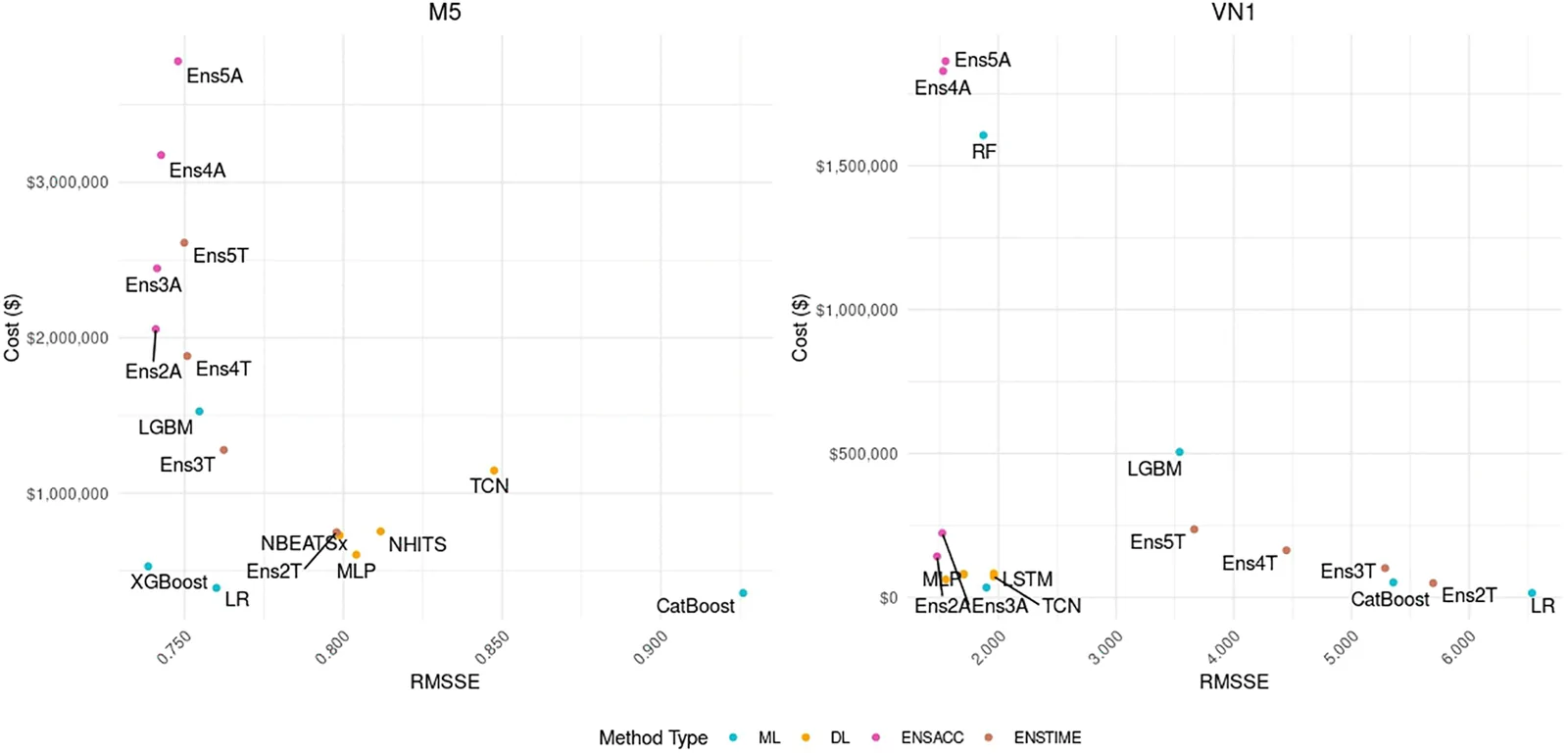
Cost analysis. RMSSE vs Cost ($) for the M5 and VN1 datasets.

**Fig 7:**
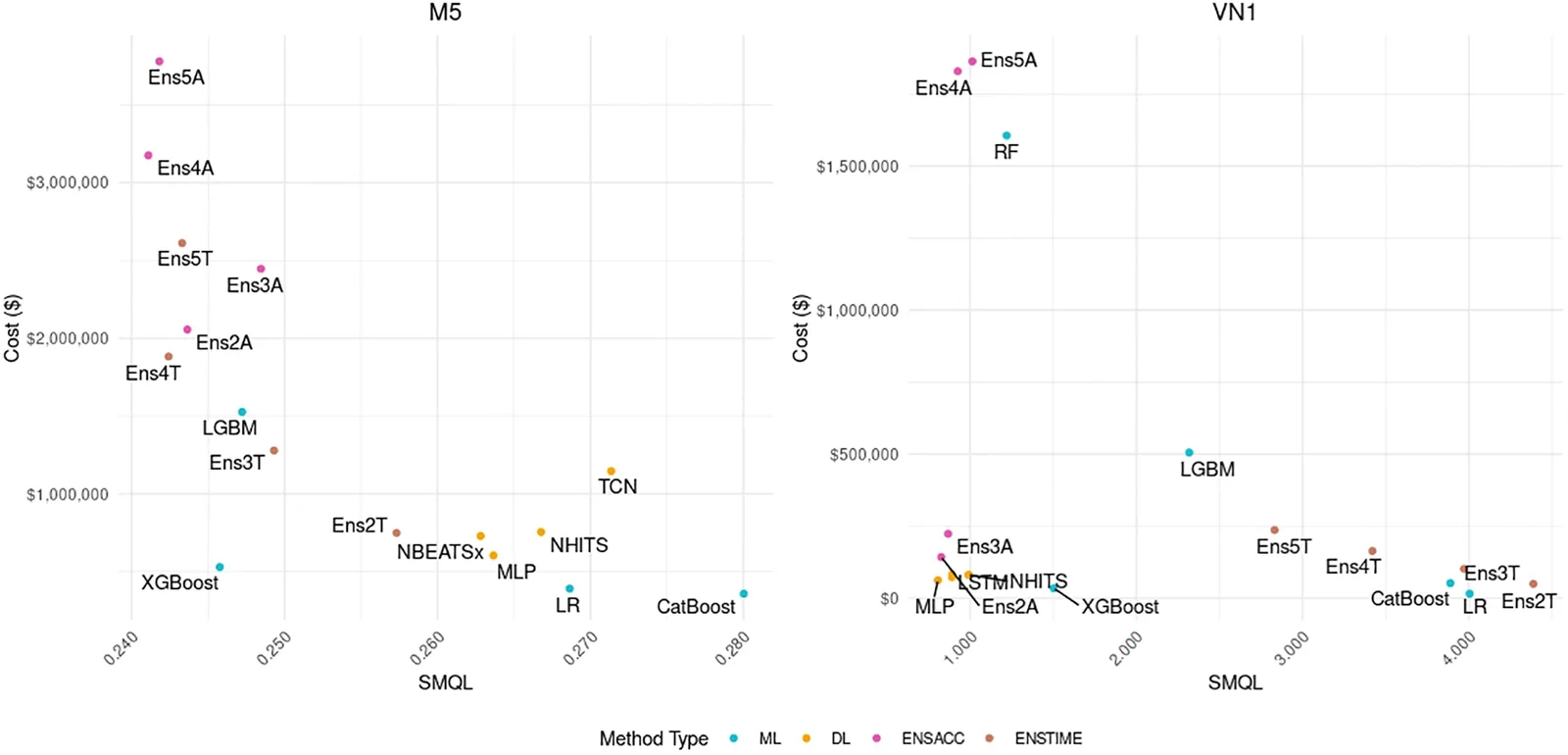
Cost analysis. SMQL vs Cost ($) for the M5 and VN1 datasets.

The total forecast production cost (Fig 5), computed by combining training time and forecast generation under a cloud-computing pricing model, highlights the trade-off central to our study. ENSACC ensembles incur the highest costs, rapidly reaching millions of dollars under frequent retraining. In contrast, ENSTIME ensembles offer a more sustainable alternative, reducing costs by over 50% in many cases. Moreover, as dataset size increases, the trade-off between forecast accuracy and cost becomes significantly more pronounced. This is clearly illustrated in Figs 6 and 7. For the VN1 dataset, ENSACC methods offer noticeable improvements in both point and probabilistic forecasting accuracy compared to most individual base models, while incurring only a modest increase in cost. In this context, there is little reason to favor ENSTIME ensembles over ENSACC ones, as the cost difference is minor and the performance gain from accuracy-based combinations is evident. However, the situation is quite different for the M5 dataset. Here, ensemble models, particularly those optimized for accuracy, are significantly more expensive than individual models. Additionally, the performance advantage of ENSACC ensembles over the most accurate base models is minimal in terms of both RMSSE and SMQL. In contrast, ENSTIME ensembles can achieve comparable levels of accuracy with substantially lower additional cost, making them a more attractive option. It is also clear that increasing the number of models in an ensemble does not necessarily lead to proportional gains in forecasting accuracy, which raises questions about the cost-effectiveness of larger combinations. Notably, forecast combinations that disregard the computational efficiency of their base models can lead to excessive and unjustified costs. This is particularly evident in the case of Ens4A and Ens5A. For instance, on the VN1 dataset, where the inclusion of Random Forest (a highly expensive model in this context) results in disproportionately high forecasting costs with only marginal accuracy improvements.

Finally, Figs 8 and 9 shows the comparison between base models and ensembles across all retraining scenarios. As expected, forecasting costs decrease exponentially with less frequent retraining. Across both datasets, ENSTIME ensembles consistently incur lower costs than their ENSACC counterparts. Moreover, the cost-saving benefits of reduced retraining frequency become more pronounced as dataset size increases. In the VN1 dataset, cost differences between retraining scenarios remain relatively small, even for ensemble models, suggesting limited sensitivity to retraining frequency. In contrast, the M5 dataset exhibits steady, incremental reductions in cost as retraining becomes less frequent.

**Fig 8:**
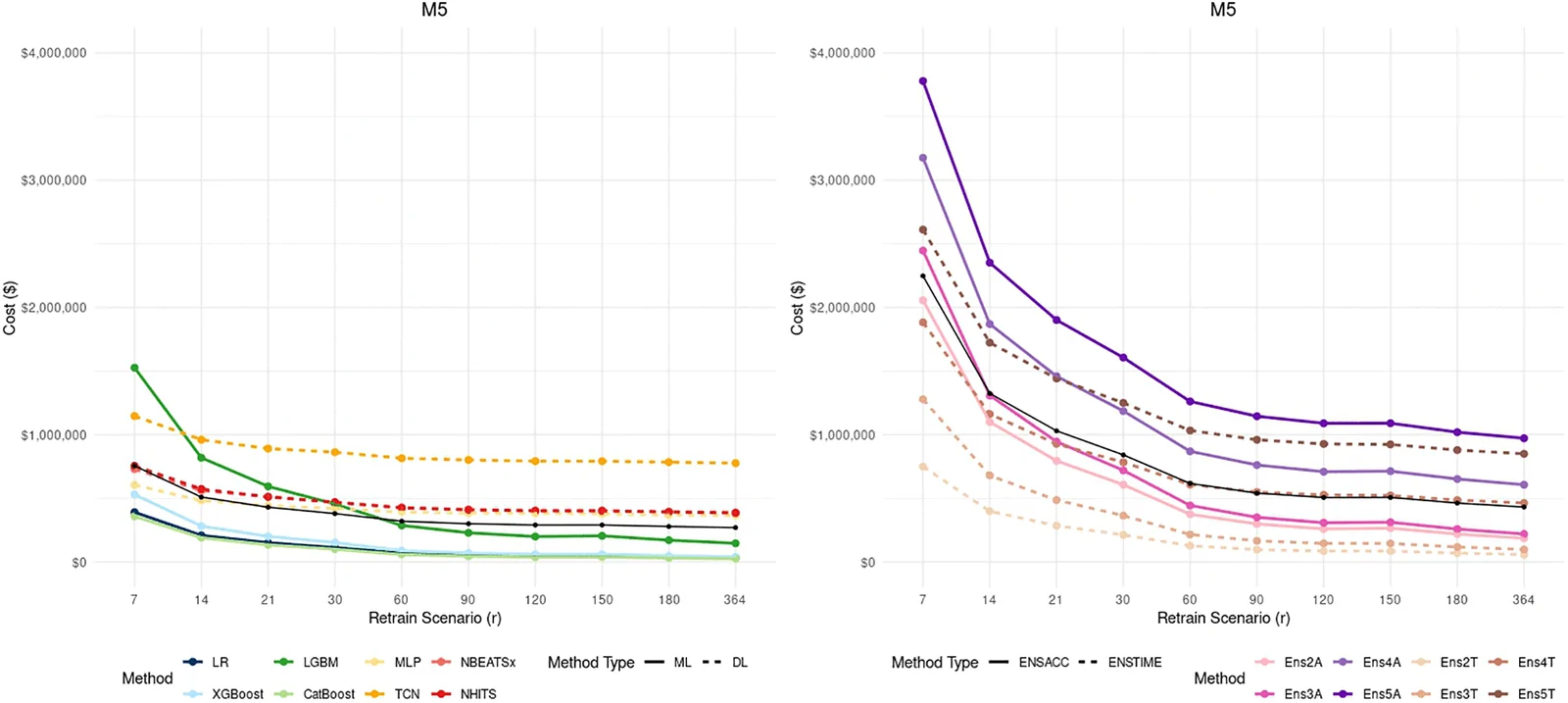
Cost analysis. Comparison between base models and ensembles for the M5 dataset. Each method and retrain scenario combination are shown in relative terms with respect to the baseline scenario, *r* = 7.

**Fig 9:**
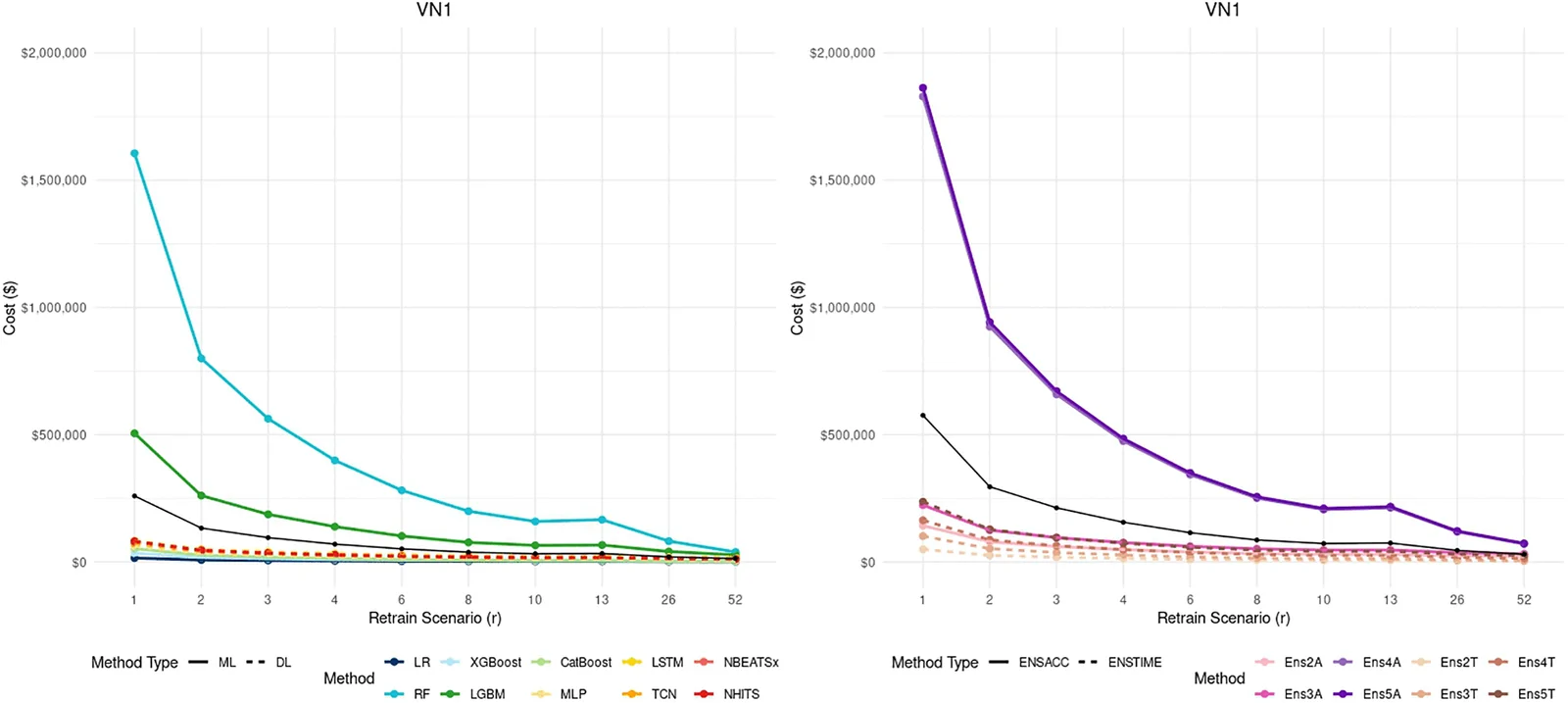
Cost analysis. Comparison between base models and ensembles for the VN1 dataset. Each method and retrain scenario combination are shown in relative terms with respect to the baseline scenario, *r* = 1.

For example, in the M5 setting, the average cost of continuous retraining for ensemble models is approximately $2,250,000, more than triple the average cost of individual base models. When retraining is entirely eliminated, this figure drops to around $500,000, representing a cost reduction of over 75%. However, even in the no-retraining scenario, ensemble models remain roughly twice as expensive as the average base model. Interestingly, the cost gap between ENSACC and ENSTIME ensembles narrows for smaller ensembles (e.g., those with two or three base models) as retraining frequency decreases. This indicates that with less frequent retraining, accuracy-based combinations (ENSACC) become increasingly comparable to efficiency-based ones (ENSTIME) in terms of forecasting cost. Thus, infrequent retraining can help align performance-optimized ensemble strategies with more budget-conscious forecasting objectives.

One might argue that, for a large retailer, these costs (and the potential savings) are relatively small. However, it’s important to emphasize that the cost reductions achieved through a combination of less frequent retraining, smaller ensemble sizes, and more efficient model combinations typically come with no loss in forecasting accuracy, particularly for point forecasts. This makes such strategies both economically and operationally attractive. These results further support our findings. ENSACC ensembles dominate in accuracy but at a significant computational premium. ENSTIME ensembles represent a middle ground. Importantly, ensembles of just two or three models (e.g., ENS2A, ENS3T) often achieve near-optimal performance, providing a compelling trade-off between complexity, cost, and accuracy, and lowering the retraining frequency can be a good strategy to improve this trade-off even in the context of ensemble learning. This comprehensive analysis reinforces the core message of the article: ensemble methods offer strong accuracy benefits, but their cost can be substantial, especially under frequent retraining. Balancing accuracy and sustainability requires deliberate model selection and retraining design, with lightweight ensembles, like ENSTIME, being a possible solution in large-scale forecasting systems.

## Conclusions

This study investigated the cost-effectiveness of ensemble learning in global time series forecasting, focusing on the interplay between forecast accuracy, computational efficiency, and retraining strategies. We evaluated ten global forecasting models, spanning both classical machine learning and deep learning techniques, alongside eight ensemble configurations, across two large-scale and industry-relevant datasets: the M5 and VN1 retail forecasting datasets. By systematically comparing different ensemble sizes, selection criteria (accuracy-based vs. efficiency-based), and retraining frequencies, our aim was to assess whether the accuracy gains of ensemble methods justify their additional computational cost, particularly in large-scale operational settings.

Our findings showed that ensemble models consistently improve both point and probabilistic forecasting performance over most individual base models. This improvement is also relevant for probabilistic accuracy, where ensembles provide better uncertainty quantification through the aggregation of diverse quantile predictions. The results support the notion that ensemble learning offers a robust mechanism for improving predictive reliability and mitigating individual model weaknesses, even in the context of global modeling. However, these benefits come at a cost. Ensemble methods are substantially more computationally intensive than single-model approaches, particularly when composed of complex or resource-heavy algorithms such as LightGBM or Random Forest. These computational demands translate directly into higher forecasting costs under cloud-based computing environments.

Our analysis further distinguished between two ensemble design philosophies: ENSACC (accuracy-based) and ENSTIME (efficiency-based). While ENSACC ensembles generally deliver higher accuracy, especially when composed of two or three strong base models, they are also more expensive. In contrast, ENSTIME ensembles, composed of lightweight, computationally efficient models, can achieve comparable accuracy at a much lower cost, making them an attractive compromise in budget-sensitive applications. Most importantly, we find that increasing the number of models in an ensemble does not always yield proportional accuracy gains and often results in rapidly escalating costs. Small ensembles (with two or three base models) are often sufficient to capture most of the benefit in accuracy.

Crucially, our study explored the effects of retraining frequency on ensemble performance and cost. Even if these effects are more pronounced for the base models alone, we showed that less frequent retraining can reduce computational time, without compromising point forecast accuracy, also in the context of ensembles. In many scenarios, especially for relatively stable datasets, even the no-retraining setup performed nearly as well as continuous retraining, confirming and extending the findings from previous work on global models [20]. Probabilistic forecasting accuracy is more sensitive to retraining, especially for higher-frequency datasets like M5, but performance declines remain modest for moderate retraining intervals. These insights are highly relevant for real-world forecasting systems, where minimizing operational cost without sacrificing accuracy is essential. Indeed, organizations can achieve economic benefits by optimizing their retraining strategies without compromising the forecast quality.

From a practical standpoint, our findings offer several actionable guidelines for forecasters and organizations deploying large-scale forecasting systems. First, ensembles should be kept small and strategically designed, prioritizing accuracy or efficiency depending on the business context. Second, frequent retraining is often unnecessary. Overall, monthly retraining emerges as a practical compromise for balancing probabilistic forecast accuracy with computational costs. However, if the primary forecasting objective is point prediction, even less frequent retraining intervals can be adopted without significantly affecting performance. Third, ENSTIME strategies represent a viable, low-cost pathway to ensemble forecasting, especially when forecast robustness is desired without substantial computational investment. Retailers and other large-scale forecasters can leverage these insights to build forecasting systems that are both accurate and sustainable. Indeed, these findings also carry broader implications for the sustainability of cross-learning driven forecasting systems. Reducing the frequency of retraining not only lowers operational costs but also leads to significant energy savings, thereby enhancing the environmental sustainability of forecasting processes. This aligns with the principles of *Green AI*, which advocates for the responsible use of computational resources to minimize the ecological footprint of machine learning applications.

While our findings provide strong evidence on the effects of ensembling global forecasting models, some limitations remain. First, we restricted our experiments to two retail datasets, which, while comprehensive and realistic, may not capture the full diversity of time series behaviors found in other domains such as finance, health care, or energy. Additionally, this study operates under the assumption that the data-generating process remains stable, without notable trends or concept drift. However, in many real-world scenarios, this assumption may not hold, potentially affecting the reliability of less frequent retraining strategies. Studying the effect of concept drift on the retraining frequency of global forecasting models is indeed a promising area of future research. Moreover, our ensemble strategies used simple averaging without dynamic weighting or more sophisticated stacking techniques, which may further improve performance at the cost of added complexity and computation time. We also assumed static cost estimates for computational resources, whereas real-world costs may vary based on cloud infrastructure, parallelism, or vendor-specific pricing. We also deliberately focused on a global modeling approach only, but local or hybrid frameworks may offer different cost-accuracy trade-offs worth exploring. Finally, we encourage further studies to explore the possible trade-off between cost and forecast stability in the context of ensemble learning. In fact, the stability of forecasts is a highly relevant yet underexplored topic, with important implications for model selection in time series forecasting.

In conclusion, our study reinforces the value of ensemble forecasting in global modeling, showing that well-designed ensemble strategies, particularly those leveraging periodic retraining and lightweight components, can deliver high accuracy at a fraction of the computational cost. Striking the right balance between accuracy, efficiency, and retraining frequency is key to deploying scalable and sustainable forecasting systems in practice.

## Supporting information

S1 File Appendix, tables, and figures supporting the results. (PDF)
