# Supplementary material for: The cost of ensembling: Is it always worth combining global retail forecasting models?
Source: PLoS One. 2026 Sep 29;21(9):e0336281. doi: 10.1371/journal.pone.0336281 (PMC13623108; doi:10.1371/journal.pone.0336281)
Supplement: Supplementary file 1 [file pone.0336281.s001.pdf]

Supporting information

S1 Appendix. Conformal inference The Conformal Inference framework introduced by (1) offers a flexible tool for uncertainty quantification, and has been successfully applied to time series forecasting problems (2). Since most of the machine learning and deep learning models we use do not natively produce probabilistic outputs, we employed Conformal Inference to generate prediction intervals. Conformal Inference is a versatile framework that quantifies predictive uncertainty using a calibration (validation) set, without relying on strong distributional assumptions (1). Originally developed for independently and identically distributed data, it has recently been adapted also to time series contexts (2). Its properties, distribution-free, model-agnostic, efficient, and suitable for small datasets, make it especially well-suited for forecasting benchmarks involving diverse models and datasets. An alternative approach is to train models using quantile loss functions to directly produce quantile forecasts. While this method can effectively capture uncertainty, as demonstrated in the M5 Uncertainty competition (3; 4), it requires training a separate model for each quantile level. This significantly increases the computational burden, making it less suitable for large-scale forecasting applications where efficiency and sustainability are critical considerations. Moreover, although multi-quantile loss functions can generate multiple quantiles and the corresponding prediction intervals, the resulting intervals are often uncalibrated, that is, the proportion of observations falling within them may not match the intended confidence level. For these multiple reasons, we chose to use Conformal Inference as a general framework for producing quantile forecasts. To ensure valid estimation of predictive uncertainty, conformal prediction intervals were computed on validation sets at least twice the length of the forecast horizon. We used a validation set four times larger than the forecast horizon for the M5 dataset and double the forecast horizon for the VN1 dataset. This limited the number of series from 30,490 to 28,298 for the M5 dataset.

S1 Table Main hyperparameter settings for machine learning forecasting models for M5 — VN1 datasets.

Model	Learning rate	Feature frac.	N. Estimators	Loss
Random Forest	0.03	0.85	300	MSE
XGBoost	0.03	0.85	500	Tweedie
LightGBM	0.03	0.85	500	Tweedie
CatBoost	0.03	—	500	Tweedie

S2 Table Main hyperparameter settings for deep learning forecasting models for M5 — VN1 datasets.

Model	Input size	Learning rate	Epochs	Scaler	Batch size
MLP	112 — 26	0.001	1000	Robust	128
LSTM	112 — 26	0.001	1000	Robust	128
TCN	112 — 26	0.001	1000	Robust	128
NBEATS	112 — 26	0.001	1000	Robust	128
NHITS	112 — 26	0.001	1000	Robust	128

S1 Fig. Friedman-Nemenyi test results based on RMSSE. The baseline scenarios are $r = 7$ for the M5 dataset and $r = 1$ for the VN1 dataset.

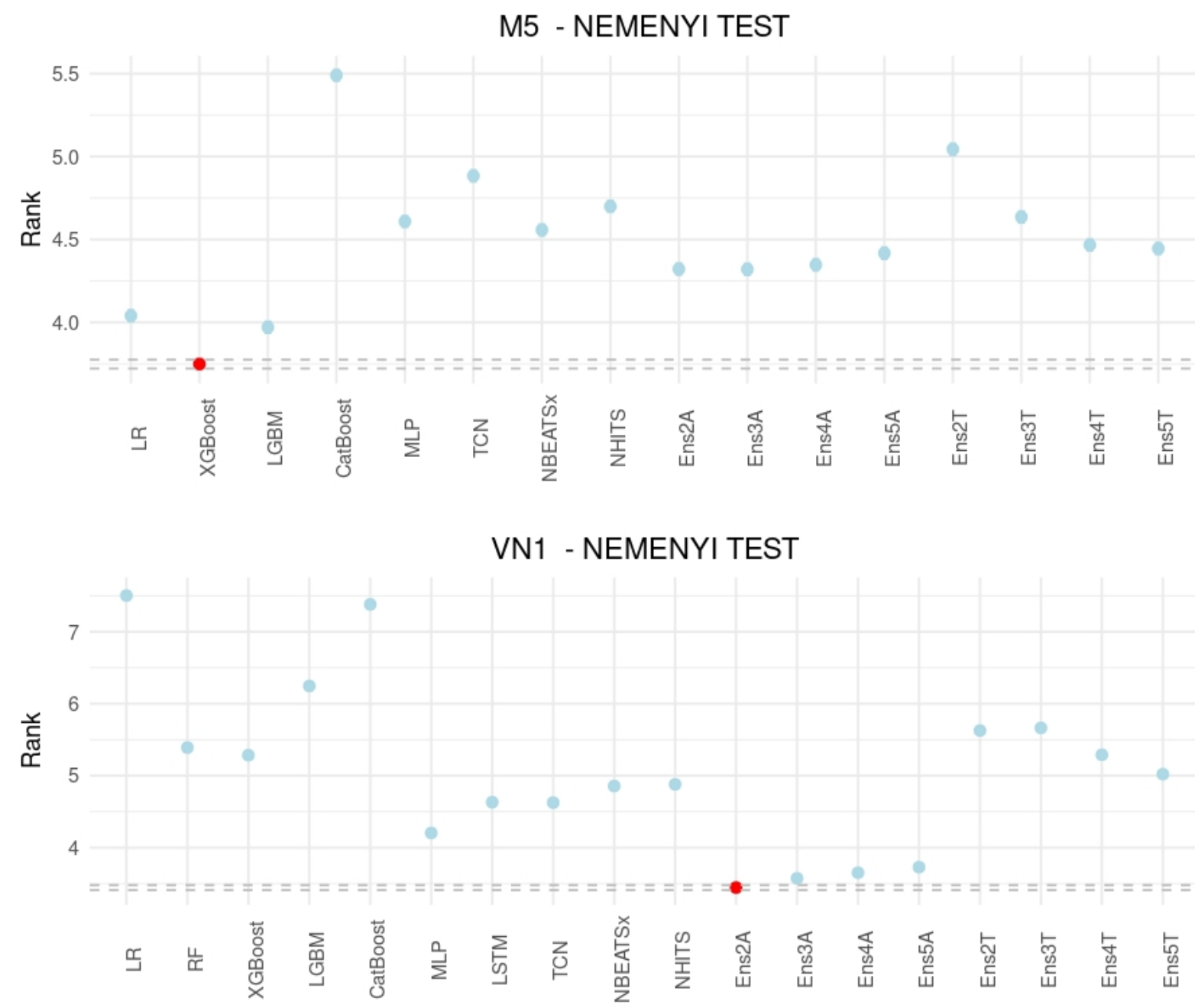

S2 Fig. Friedman-Nemenyi test results based on SMQL. The baseline scenarios are $r = 7$ for the M5 dataset and $r = 1$ for the VN1 dataset.

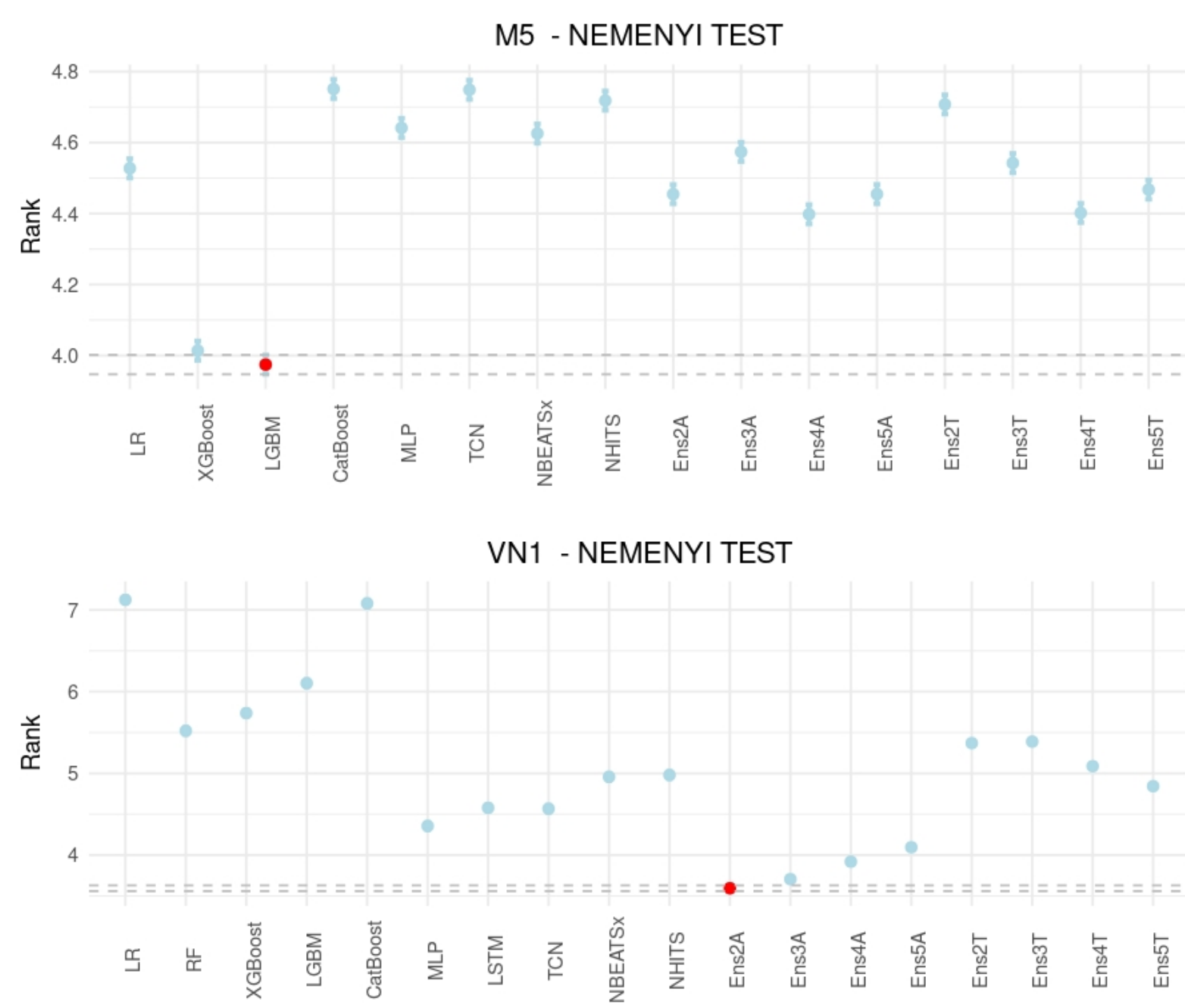

S3 Fig. M5 Friedman-Nemenyi test results based on RMSSE.

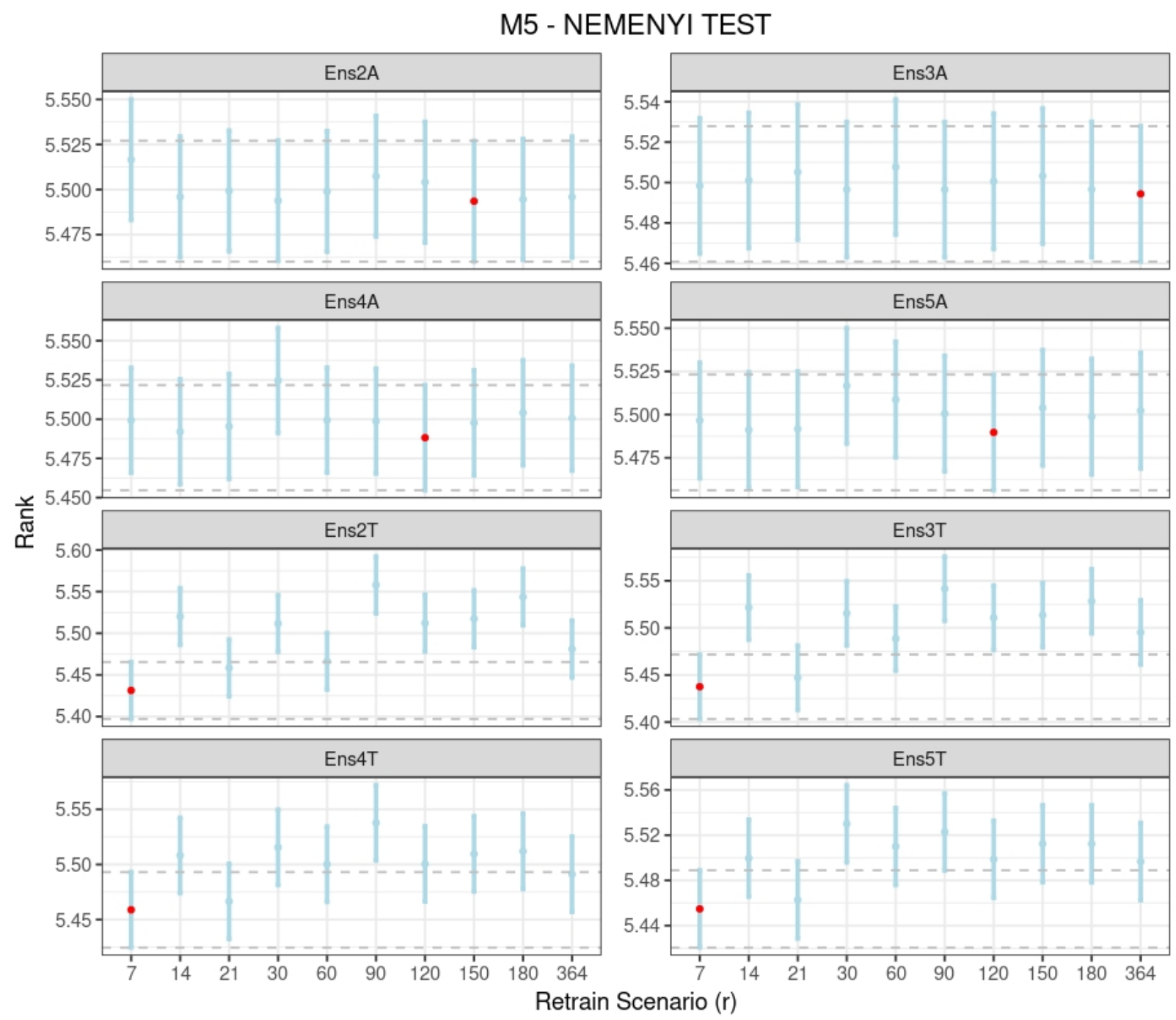

S4 Fig. VN1 Friedman-Nemenyi test results based on RMSSE.

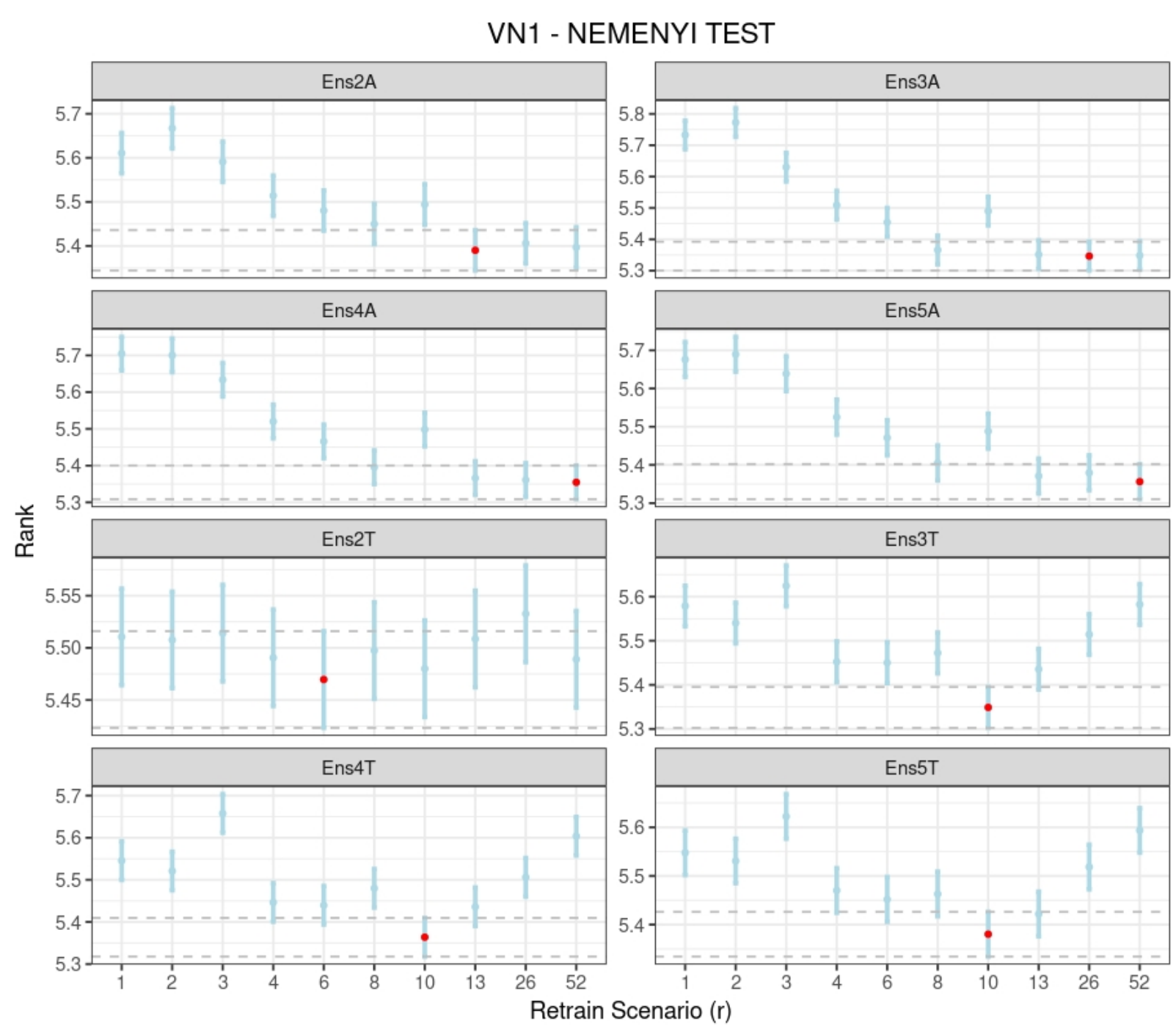

S5 Fig. M5 Friedman-Nemenyi test results based on SMQL.

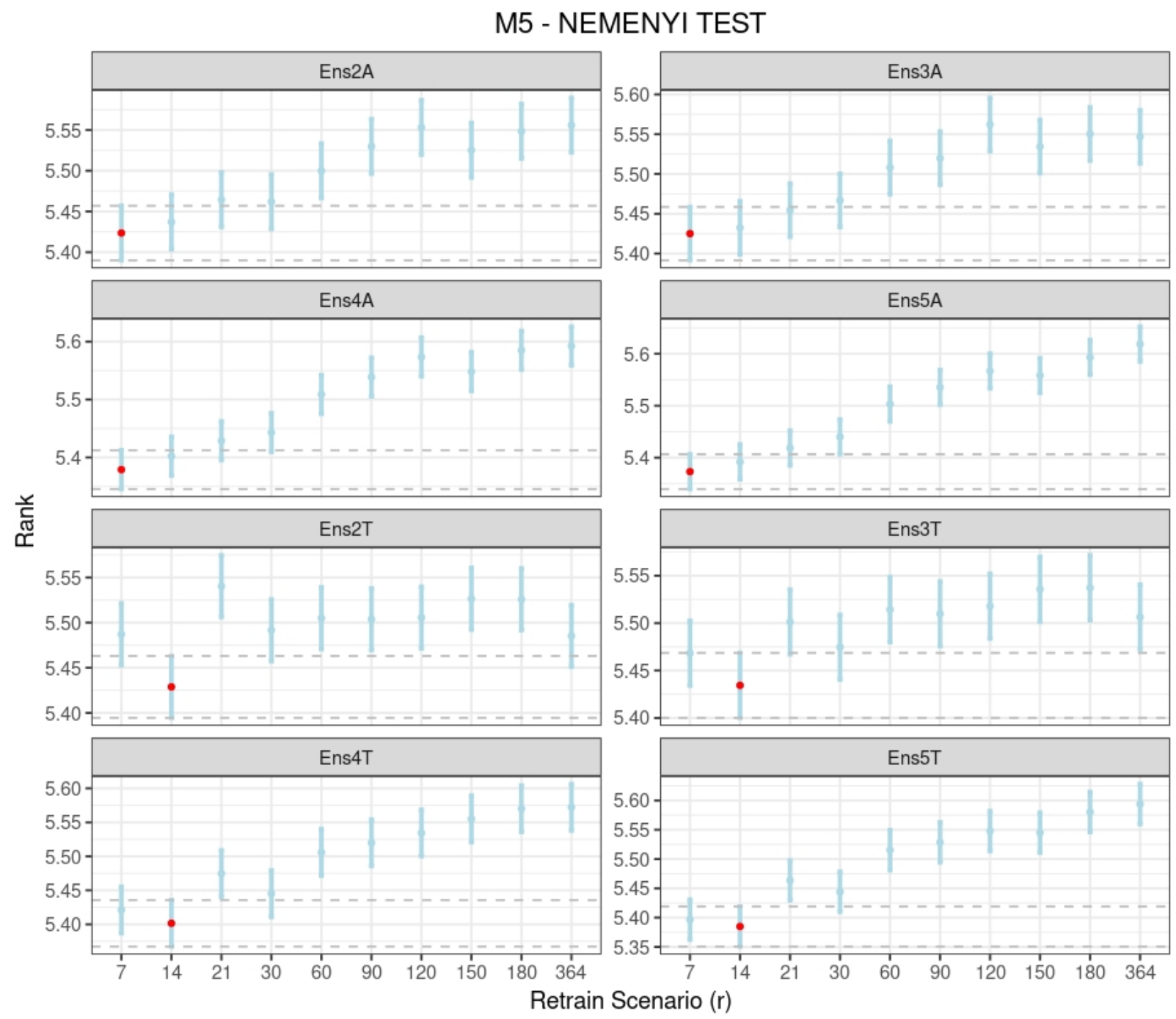

S6 Fig. VN1 Friedman-Nemenyi test results based on SMQL.

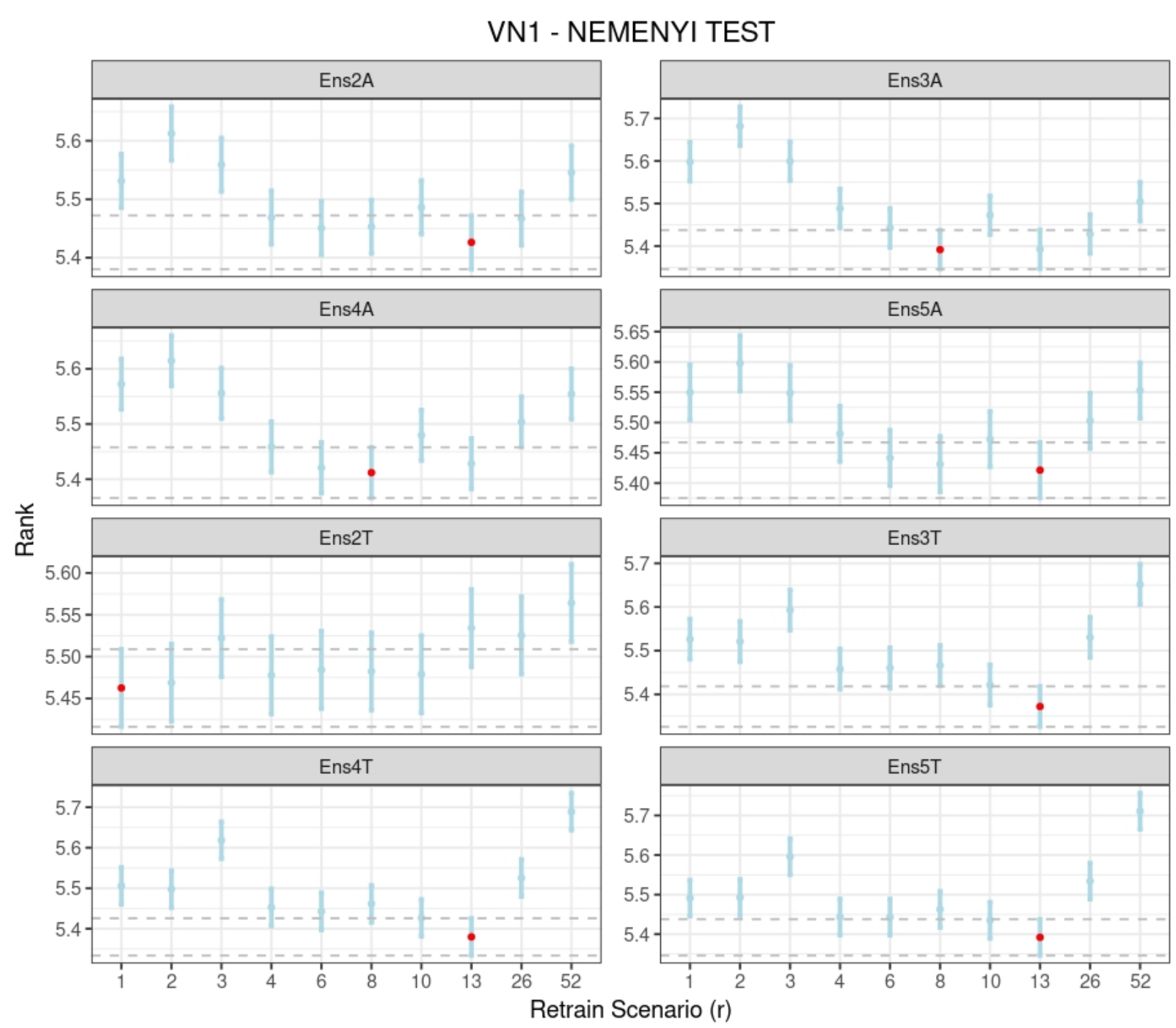

S7 Fig. Experimental design pipeline.

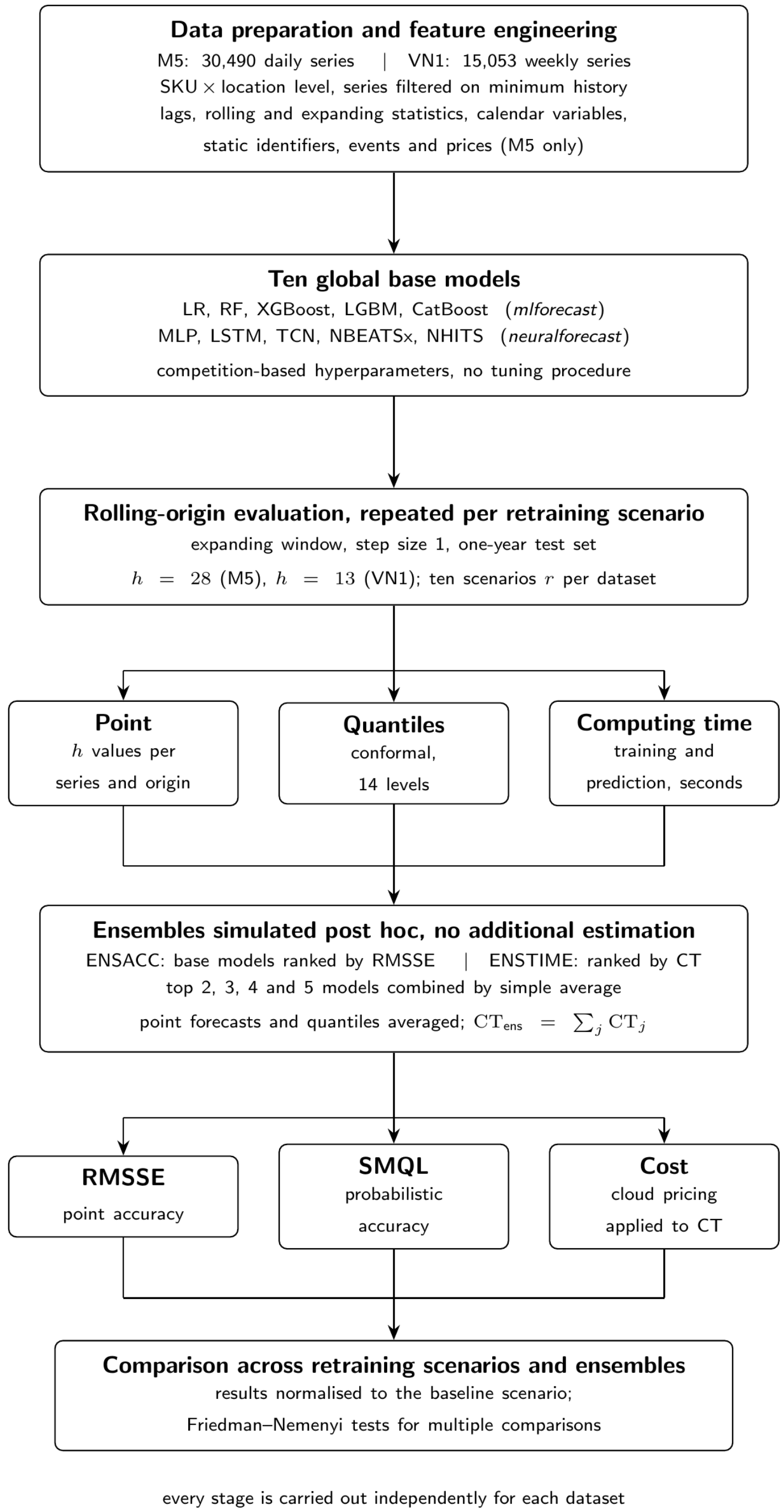

References

1. Vovk V, Gammerman A, Shafer G. Algorithmic Learning in a Random World. Berlin, Heidelberg: Springer-Verlag; 2005.

2. Stankeviciute K, M Alaa A, van der Schaar M. Conformal Time-series Forecasting. In: Ranzato M, Beygelzimer A, Dauphin Y, Liang PS, Vaughan JW, editors. Advances in Neural Information Processing Systems. vol. 34. Curran Associates, Inc.; 2021. p. 6216-28. Available from: https://proceedings.neurips.cc/paper_files/paper/2021/file/312f1ba2a72318edaaa995a67835fad5-Paper.pdf.

3. Makridakis S, Spiliotis E, Assimakopoulos V, Chen Z, Gaba A, Tsetlin I, et al. The M5 uncertainty competition: Results, findings and conclusions. International Journal of Forecasting. 2022;38(4):1365-85. Special Issue: M5 competition. Available from: https://www.sciencedirect.com/science/article/pii/S0169207021001722. doi:https://doi.org/10.1016/j.ijforecast.2021.10.009.

4. Lainder AD, Wolfinger RD. Forecasting with gradient boosted trees: augmentation, tuning, and cross-validation strategies: Winning solution to the M5 Uncertainty competition. International Journal of Forecasting. 2022;38(4):1426-33. Special Issue: M5 competition. Available from: https://www.sciencedirect.com/science/article/pii/S0169207021002090. doi:https://doi.org/10.1016/j.ijforecast.2021.12.003.
