# Supplementary material for: The cost of ensembling: Is it always worth combining global retail forecasting models?
Source: PLoS One. 2026 Sep 29;21(9):e0336281. doi: 10.1371/journal.pone.0336281 (PMC13623108; doi:10.1371/journal.pone.0336281)
Supplement: Supplementary file 2 [file pone.0336281.s003.pdf]

May 18, 2026

Editor-in-Chief
PLOS One
Elsevier

Dear Editor,

We want to thank you and the reviewers for the careful reading of our manuscript and the constructive feedback provided. We have carefully revised the paper to address all the comments and suggestions.

We prepared a detailed response to each reviewer's comment, addressing their specific requests and clarifying our methodological choices.

Response to Editors

We sincerely thank all editors for their constructive feedback. Below, we address each comment point by point.

- We have carefully reviewed the manuscript and supplementary files to ensure compliance with PLOS ONE formatting and submission requirements.
- Thank you for highlighting the journal's code sharing requirements. All author-generated code that underpins the findings of the manuscript is already publicly available on GitHub and released under an open-source license. The repository link https://github.com/marcozanotti/global-retrain.
- Thank you for pointing this out. We have carefully reviewed the Funding Information and Financial Disclosure sections and corrected them so that the information is now fully consistent across the submission.
- Thank you for the clarification. All datasets used in this study are publicly available and can be freely shared. We have revised the Data Availability Statement accordingly to make this explicit and to ensure full compliance with the journal's open data policy.

Response to Reviewers

We sincerely thank all reviewers for their constructive feedback. Below, we address each comment point by point. Reviewer comments are in *italics*, followed by our responses.

Reviewer 1

The paper presents an interesting approach that evaluated ten base models and eight ensemble configurations across two large-scale retail datasets, considering both point and probabilistic accuracy under varying retraining frequencies. The analysis is interesting in the area of forecasting.

- *I suggest the author provide more detail on the time series. While they mention having information on products and vendors, it's unclear what type of data was used in the experiments. Was it just products per week? Please specify the size of the observation window and the size of the prediction. Also, please indicate whether it's a multi-step prediction, a single-step prediction, or a multi-variable, multi-step prediction.*

We agree that additional details about the structure of the time series and the forecasting setup improve the clarity and reproducibility of the study. In the revised manuscript, we have expanded the description of the datasets and the forecasting task. Specifically, we clarified that the experiments are conducted on large-scale retail datasets composed of daily (M5 dataset) and weekly (VN1 dataset) sales time series at the product level (SKU). We now explicitly report in a new table (Table 1): the length of the historical observation window used by the models, the forecast horizon, and the type of forecasting task. We also clarified that the problem is formulated as a global multi-series, multi-step forecasting task, where models are trained jointly across many product-level time series and generate forecasts for the entire prediction horizon.

- *The author mentions using Nixtla. I'm a little concerned about the study's reproducibility, given that Nixtla is a paid service. How can reproducibility be guaranteed when using these platforms, which may not be accessible to everyone?*
  We thank the reviewer for raising this important point regarding reproducibility. We would like to clarify that Nixtla provides both proprietary services (e.g., TimeGPT) and fully open-source libraries. In this study, we exclusively used Nixtla's open-source libraries, which are freely accessible and publicly available to anyone. The models used in the experiments are standard forecasting methods implemented in Nixtla's open-source ecosystem (e.g., StatsForecast, MLForecast, NeuralForecast). Therefore, the experimental pipeline does not rely on any proprietary service or paid infrastructure.

- *According to the Nixtla platform used, the model training and evaluation apparently involved using prompts with LLM. It is suggested that the author provide further explanation regarding whether this training was indeed done at the prompt level, in order to validate the accuracy of the results.*
  We thank the reviewer for this comment and the opportunity to clarify a potential misunderstanding. Nixtla provides two different types of tools: TimeGPT, a proprietary LLM-based forecasting service that uses prompting; Open-source forecasting libraries (e.g., StatsForecast, MLForecast, NeuralForecast), which implement classical statistical, machine learning, and deep learning forecasting methods. In this study, we did not use TimeGPT or any LLM-based or prompt-based methodology. All experiments were conducted using only the open-source Nixtla libraries, which rely on conventional machine learning and deep learning models for time series forecasting. Therefore: no prompting was used, no large language model was involved, and model training followed standard supervised learning procedures using historical time series data.

- *The author mentions that the parameters were selected from those used in the competitions. It would be ideal to include a table with these parameters for each model. Furthermore, could the author clarify whether a hyperparameter search was also conducted and what strategies were used?*
  We thank the reviewer for this helpful suggestion. We agree that additional clarity regarding model configurations improves reproducibility and transparency. We have now included a dedicated table reporting the main hyperparameter settings used for each model in the Supplementary information. Regarding hyperparameter optimization, no additional tuning was performed in this study. This decision was driven by computational budget constraints, as our experimental design involves repeated retraining across multiple models and datasets, making full hyperparameter optimization prohibitively expensive. Instead, we relied on established hyperparameter configurations from high-performing solutions in forecasting competitions, which have been shown to be competitive benchmarks in similar settings.

- *The author should mention if the datasets had missing data, what percentage, and how they were addressed.*
  We thank the reviewer for raising this point. Both datasets used in this study, namely the M5 and VN1 competition datasets, do not contain missing values. Therefore, no imputation or additional preprocessing for missing data was required. We have clarified this point in the revised manuscript for completeness.
- *In section "Overview, the author should complete each section. For instance, the manuscript said: "After a brief review of related works (Section ?), in Section we describe the design of the experiment used in our study. The datasets and their characteristics are presented in ?, while ..." "Finally, Section ? contains our summary and conclusions." Same situation for line 194.*
  We thank the reviewer for carefully identifying these issues. We have corrected all missing and inconsistent section references throughout the manuscript, including those in the Overview section and line 194. The revised version now includes complete and accurate section numbering.
- *In Table 1, it is suggested to add the description of each acronym at the bottom of the table.*
  We thank the reviewer for this suggestion. We have updated Table 1 to improve clarity and readability.
- *Acronyms must be described first (e.g. IA, ENSACC, i.i.d).*
  We thank the reviewer for this remark. We have revised the manuscript to ensure that all acronyms are defined at their first occurrence in the text (e.g., IA, ENSACC, i.i.d.), as suggested.

Reviewer 2

This paper studies the trade-off between forecasting accuracy and computational cost in ensemble learning for global retail demand forecasting. Using the M5 and VN1 datasets, the author evaluates a range of base models and ensemble configurations under different retraining frequencies. Both point and probabilistic performance are assessed, with computational time used as a proxy for cost. The results show that ensembles generally improve accuracy, especially for probabilistic forecasts, but at a higher computational cost, particularly for accuracy-driven combinations. The paper also finds that small ensembles (2–3 models) are often sufficient, and that less frequent retraining can substantially reduce cost with little loss in accuracy.

The paper addresses an important and timely problem, as managing the cost–accuracy trade-off is increasingly relevant in large-scale forecasting systems. The empirical analysis is thorough and well designed. The use of two widely studied datasets, multiple base models and ensemble strategies, and different retraining scenarios provides a solid experimental setup. The practical value of the findings is another strong point. The results offer clear guidance for practitioners, particularly regarding the benefits of small ensembles and reduced retraining frequency. The explicit link to computational cost and cloud deployment further enhances the paper's relevance.

- *Ensemble construction: The analysis is limited to simple averaging ensembles. While this is a reasonable baseline, it restricts the scope of the conclusions. More adaptive approaches (e.g., weighted or dynamic combinations) could lead to different cost–accuracy trade-offs. At a minimum, the paper should discuss how alternative ensemble strategies might affect the results. Including an additional benchmark would strengthen the analysis.*
  We thank the reviewer for this insightful suggestion. We agree that alternative ensemble strategies such as weighted averaging, dynamically adjusted combinations, and stacking-based approaches may potentially improve predictive accuracy by adapting model contributions or learning optimal combination schemes. However, these methods typically introduce additional

computational complexity, including weight estimation procedures, validation overhead, or the need for secondary learning models. Moreover, it is not always clear how to combine probabilistic forecasts with these approaches. In this study, the focus is specifically on evaluating the trade-off between forecasting accuracy and computational cost under repeated retraining scenarios. For this reason, and due to computational budget constraints, we restrict our analysis to simple averaging ensembles, which provide a strong and widely used baseline while avoiding additional optimization or training costs. Exploring more advanced ensemble strategies was not feasible within the computational budget available for this study, but it is pointed out as a limitation and direction for future research. We have added a corresponding discussion in the revised manuscript to clarify this design choice and to acknowledge that more sophisticated ensemble methods may lead to different cost–accuracy trade-offs, albeit at higher computational expense.

- *Simplified cost modeling: The cost analysis relies on a simplified setup with fixed cloud pricing and a single hardware configuration. This does not fully reflect real-world systems, where parallelization and different pricing schemes can significantly affect costs. The author should make these assumptions explicit and discuss their implications, as the current setup may overstate the cost disadvantage of ensembles.*
  We thank the reviewer for this important observation. We agree that real-world forecasting systems may involve a wide range of computational configurations, including different hardware setups, pricing schemes, and levels of parallelization, all of which can influence absolute cost estimates. However, it is not feasible to exhaustively evaluate all possible system configurations within a single study. For this reason, we adopt a controlled experimental design with a fixed hardware configuration and constant pricing scheme in order to ensure comparability across models and retraining scenarios. This approach follows the design adopted in prior work on retraining in forecasting, in particular *Spiliotis E, Petropoulos F. On the update frequency of univariate forecasting models. European Journal of Operational Research*, which is, to the best of our knowledge, the only existing study that explicitly investigates retraining frequency in forecasting systems under a cost-aware perspective. That study also relies on a single hardware configuration and fixed cost assumptions to maintain experimental consistency. We have clarified these assumptions explicitly in the revised manuscript and added a discussion on their implications. In particular, we acknowledge that while this setup enables a controlled comparison of relative computational costs, it may not capture all real-world optimizations such as parallel execution or heterogeneous pricing environments, which could affect absolute cost levels but are unlikely to change the qualitative conclusions regarding the cost–accuracy trade-offs.

- *Statistical analysis: Although the Friedman–Nemenyi test is mentioned, the results are not reported or discussed. Most conclusions rely on descriptive comparisons, despite the fact that performance differences are often small. Stronger statistical support is needed, especially for claims about the near-optimality of small ensembles and the similarity between methods.*
  We thank the reviewer for this valuable suggestion. We agree that stronger statistical support improves the robustness of the conclusions. In response, we have now included the results of the Friedman–Nemenyi test in the Supplementary information. These results provide a formal statistical assessment of the performance differences among the compared methods and support the conclusions drawn in the manuscript.

- *Data stability assumption: The analysis assumes relatively stable data, with limited discussion of concept drift or trends. This is a strong assumption in retail forecasting, where demand can change abruptly. The conclusion that less frequent retraining does not harm performance should therefore be interpreted cautiously. The authors should clarify under which conditions this result*

is expected to hold.

We thank the reviewer for this important observation. We agree that retail demand forecasting can be affected by non-stationarities and concept drift, where abrupt changes in demand patterns may occur due to external events, seasonality shifts, or structural breaks. In our study, the empirical analysis is based on two large-scale retail datasets (M5 and VN1) that already incorporate substantial heterogeneity across products, stores, and time, including intermittent demand and varying seasonal patterns. However, we acknowledge that our experimental setup does not explicitly isolate or model concept drift mechanisms, and therefore implicitly assumes a degree of temporal stability over the evaluation horizon. For this reason, it is explicitly stated that "This robustness likely stems from the relative stability of the datasets, which do not exhibit substantial structural changes or concept drift. Under such conditions, base models continue to capture underlying demand patterns effectively over time, and ensemble combinations further enhance stability as retraining becomes less frequent.". We have revised the manuscript to clarify that the conclusion regarding the limited impact of reduced retraining frequency should be interpreted under conditions of moderate and gradual data evolution, as observed in the considered datasets. In highly non-stationary environments, where abrupt structural changes or strong regime shifts occur, more frequent retraining may still be necessary to maintain optimal performance. This clarification has been added to the discussion section to better delineate the scope of validity of our findings. Moreover, it is pointed out as a limitation and future research.

- *In the final paragraph of the "Related Works" section, the manuscript claims to offer both theoretical clarity and practical guidance. However, the contribution of the paper is predominantly practical in nature.*
  We thank the reviewer for this insightful observation. We agree that the main contribution of the paper is primarily empirical and practical, focusing on experimental evidence and applied insights. We have revised the final paragraph of the "Related Works" section to better reflect this emphasis and to avoid overstating the theoretical contribution of the study.
- *The analysis of Figure 7 in section "Results and Discussion" is confusing.*
  We thank the reviewer for pointing this out. The issue was due to a missing figure for the VN1 dataset in the original version of the manuscript, which led to incomplete and potentially confusing interpretation of Figure 7. This has now been corrected in the revised manuscript by including the missing VN1 figure. The section is now complete.
- *Some formatting errors need correction, e.g., missing section references.*
  We thank the reviewer for carefully identifying these issues. We have corrected all missing and inconsistent section references throughout the manuscript.
- *Table 4 is quite dense and could better highlight key trade-offs.*
  We thank the reviewer for this helpful suggestion. We agree that the original version of Table 4 was dense due to the simultaneous presentation of multiple metrics, model families, and ensemble configurations. To improve readability and better highlight the key accuracy–computational cost trade-offs, we have revised the presentation of the table. Otherwise, we will need more space to separate the single table into many smaller ones.

We believe that these revisions substantially improve the manuscript, making its contributions clearer, more rigorous, and better aligned with the expectations of the readership. We are grateful for the reviewers' insightful feedback, which has helped us to strengthen our work.

Thank you for your consideration. We look forward to your response.

Sincerely,
