# Supplementary material for: The cost of ensembling: Is it always worth combining global retail forecasting models?
Source: PLoS One. 2026 Sep 29;21(9):e0336281. doi: 10.1371/journal.pone.0336281 (PMC13623108; doi:10.1371/journal.pone.0336281)
Supplement: Supplementary file 3 [file pone.0336281.s004.pdf]

August 11, 2026

Editor-in-Chief
PLOS One
Elsevier

Dear Editor,

We want to thank you and the reviewers for the careful reading of our manuscript and the constructive feedback provided. We have carefully revised the paper to address all the comments and suggestions.

We prepared a detailed response to each reviewer's comment, addressing their specific requests and clarifying our methodological choices.

Response to Editors

We sincerely thank all editors for their feedback. We addressed all the points raised by Reviewer 1.

Response to Reviewers

We sincerely thank all reviewers for their constructive feedback. Below, we address each comment point by point. Reviewer comments are in *italics*, followed by our responses.

Reviewer 1

Dear author, thank you for addressing the previous comments. I consider the current version to be clear and concise. However, I have a couple of additional comments.

- *It is important to mention that the link to the GitHub repository does not exist. Could you please check it?*
  We checked the link, and now it should be publicly available.
- *The author states: "Given a historical observation window of length n, the model produces forecasts for a fixed horizon h, generating all future values recursively." Table 1 subsequently lists the values n = 4 years and h = 28. I am still wondering: is the model input a single variable? Does this variable span a 4-year period? What is the size of the time step—1 hour, 1 day, 1 week, or 1 month? Furthermore, is the model output a multi-step prediction? That is, are 28 steps generated for a single variable? Would these steps represent hours, days, or months? I understand that M5 has a horizon of 28 and VN1 has one of 13; But please, could you clarify what the output looks like? Specifically, the time step? I gather that the model input covers 4 years (though the time step size isn't clear to me yet) and that the output has a size of 28 (again, the time step size isn't clear). Also, I'd like to know if we are dealing with a single variable or multiple variables. Along these same lines, would it be possible for the author to provide a graph showing the input steps and the output steps? This would greatly help in classifying the generated model.*
  We thank the Reviewer for pointing this out. Re-reading the sentence, we agree it conflates three distinct quantities — the length of the training sample, the length of the input window used to build model inputs, and the forecast horizon — and that Table 1 did not state the

time step unambiguously. We answer each question in turn and have revised the manuscript accordingly.

Time step. The time step is one day for M5 and one week for VN1. The values in parentheses in Table 1 (7 and 52) were the seasonal periods, not the granularity; we have separated these into two rows to remove the ambiguity.

Single or multiple variables. The target is a single variable: unit sales (demand). Each time series records the units sold of one product at one location per time step — SKU × store for M5 (30,490 series) and product × warehouse for VN1 (15,053 series). We therefore forecast one variable, not a vector of jointly -modelled interdependent variables.

The model inputs, however, are multivariate. For each series and each forecast origin the input vector comprises: (i) lagged values of the target; (ii) rolling and expanding statistics of the target; (iii) calendar variables (day-of-week, week, month, year); (iv) static categorical identifiers (store, item, category, department, state for M5; retailer, warehouse, product for VN1); and (v) for M5, exogenous event and price/promotion variables. The setting is thus univariate forecasting with covariates, estimated globally.

What "global" means here. A single model is estimated jointly on all series of a dataset — the parameters are shared across series, and the training sample is the pooled panel of all series (Eq 1). We do not fit one model per series.

The 4 or 3 years is the training sample, not the input window. The value n in the corrected Table 1 (1,460 daily observations for M5; 157 weekly for VN1) is the length of the initial training sample available per series, from which the model is estimated. It is not the input passed to the model at prediction time. Because we use an expanding-window rolling-origin evaluation, the input window grows as the origin advances by the step size, which we set equal to 1.

Output. The output is a multi-step-ahead path for the same single target variable: h consecutive future values of unit sales, at the same granularity as the input. For M5, h = 28 corresponds to the next 28 days (four weeks); for VN1, h = 13 corresponds to the next 13 weeks (approximately one quarter). Both are business-relevant replenishment horizons in retail.

Concretely, for M5 at origin t and series i, the model returns the predicted units sold of product i at its store on days t+1, ..., t+28. With a step size of one, origins advance one day at a time across the one-year test set, yielding 336 origins per series and almost 10.3M forecast paths in total; all reported metrics are averaged over origins and series.

The newly added Figure 1 helps understand the whole process.

Recursive versus direct. We are grateful for the opportunity to correct an imprecision: the strategy is not recursive for all models. The machine learning models (mlforecast) use a recursive strategy — a one-step-ahead model applied iteratively, with predictions fed back as lags. The deep learning models (neuralforecast) use a direct multi-output strategy, emitting all h values in a single forward pass. The original sentence over-generalised the former to all methods; the revised text distinguishes them explicitly. We deliberately retained each framework's native strategy rather than forcing a common one, since our objective is to compare methods as they are actually deployed, and imposing a non-native strategy would have required re-implementing several architectures and would have distorted the computing-time comparison that is central to the study.

- *If I may, one additional suggestion is to include a diagram showing the complete pipeline; this would be ideal for understanding the applied methodology.*

  We thank the Reviewer for this suggestion. We have added (in the supplementary material) a new figure presenting the complete experimental pipeline, from data preparation through

model estimation, rolling-origin evaluation under the retraining scenarios, post-hoc ensemble construction, and the accuracy, probabilistic, and cost evaluations. Together with Fig 1, we believe this makes the applied methodology substantially easier to follow.

Reviewer 2

No comments.

We believe that these revisions substantially improve the manuscript, making its contributions clearer, more rigorous, and better aligned with the expectations of the readership. We are grateful for the reviewers' insightful feedback, which has helped us to strengthen our work.

Thank you for your consideration. We look forward to your response.

Sincerely,
