# Supplementary material for: The cost of ensembling: Is it always worth combining global retail forecasting models?
Source: PLoS One. 2026 Sep 29;21(9):e0336281. doi: 10.1371/journal.pone.0336281 (PMC13623108; doi:10.1371/journal.pone.0336281)
Supplement: Peer review history

# Peer review: Decision Letter 0

**DOI:** 10.1371/journal.pone.0336281.r001

**Authors:** Flavio Ziegelmann

12 May 2026

PONE-D-25-56430

The cost of ensembling: is it always worth combining global retail forecasting models?

PLOS One?

Dear Dr. Zanotti,

Thank you for submitting your manuscript to PLOS ONE. After careful consideration, we feel that it has merit but does not fully meet PLOS ONE’s publication criteria as it currently stands. Therefore, we invite you to submit a revised version of the manuscript that addresses the points raised during the review process.

Please submit your revised manuscript by Jun 26 2026 11:59PM. If you will need more time than this to complete your revisions, please reply to this message or contact the journal office at plosone@plos.org. When you're ready to submit your revision, log on to https://www.editorialmanager.com/pone/ and select the 'Submissions Needing Revision' folder to locate your manuscript file.

- A letter that responds to each point raised by the academic editor and reviewer(s). You should upload this letter as a separate file labeled 'Response to Reviewers'.
- A marked-up copy of your manuscript that highlights changes made to the original version. You should upload this as a separate file labeled 'Revised Manuscript with Track Changes'.
- An unmarked version of your revised paper without tracked changes. You should upload this as a separate file labeled 'Manuscript'.

If you would like to make changes to your financial disclosure, please include your updated statement in your cover letter. Guidelines for resubmitting your figure files are available below the reviewer comments at the end of this letter.

If applicable, we recommend that you deposit your laboratory protocols in protocols.io to enhance the reproducibility of your results. Protocols.io assigns your protocol its own identifier (DOI) so that it can be cited independently in the future. For instructions see: https://journals.plos.org/plosone/s/submission-guidelines#loc-laboratory-protocols. Additionally, PLOS ONE offers an option for publishing peer-reviewed Lab Protocol articles, which describe protocols hosted on protocols.io. Read more information on sharing protocols at https://plos.org/protocols?utm_medium=editorial-email&utm_source=authorletters&utm_campaign=protocols.

As the corresponding author, your ORCID iD is verified in the submission system and will appear in the published article. PLOS supports the use of ORCID, and we encourage all coauthors to register for an ORCID iD and use it as well. Please encourage your coauthors to verify their ORCID iD within the submission system before final acceptance, as unverified ORCID iDs will not appear in the published article. *Only*  the individual author can complete the verification step; PLOS staff *cannot*  verify ORCID iDs on behalf of authors.

We look forward to receiving your revised manuscript.

Kind regards,

Flavio A. Ziegelmann, Ph.D.

Academic Editor

PLOS One

Journal Requirements:

When submitting your revision, we need you to address these additional requirements.

1. Please ensure that your manuscript meets PLOS ONE's style requirements, including those for file naming. The PLOS ONE style templates can be found at

https://journals.plos.org/plosone/s/file?id=wjVg/PLOSOne_formatting_sample_main_body.pdf and

https://journals.plos.org/plosone/s/file?id=ba62/PLOSOne_formatting_sample_title_authors_affiliations.pdf

2. Please note that PLOS One has specific guidelines on code sharing for submissions in which author-generated code underpins the findings in the manuscript. In these cases, we expect all author-generated code to be made available without restrictions upon publication of the work. Please review our guidelines at https://journals.plos.org/plosone/s/materials-and-software-sharing#loc-sharing-code and ensure that your code is shared in a way that follows best practice and facilitates reproducibility and reuse.

3. We note that the grant information you provided in the ‘Funding Information’ and ‘Financial Disclosure’ sections do not match.

When you resubmit, please ensure that you provide the correct grant numbers for the awards you received for your study in the ‘Funding Information’ section.

4. Thank you for stating the following financial disclosure:

"The author(s) received no specific funding for this work."

At this time, please address the following queries:

a)        Please clarify the sources of funding (financial or material support) for your study. List the grants or organizations that supported your study, including funding received from your institution.

b)        State what role the funders took in the study. If the funders had no role in your study, please state: “The funders had no role in study design, data collection and analysis, decision to publish, or preparation of the manuscript.”

c)        If any authors received a salary from any of your funders, please state which authors and which funders.

d)        If you did not receive any funding for this study, please state: “The authors received no specific funding for this work.”

Please include your amended statements within your cover letter; we will change the online submission form on your behalf.

5. When completing the data availability statement of the submission form, you indicated that you will make your data available on acceptance. We strongly recommend all authors decide on a data sharing plan before acceptance, as the process can be lengthy and hold up publication timelines. Please note that, though access restrictions are acceptable now, your entire data will need to be made freely accessible if your manuscript is accepted for publication. This policy applies to all data except where public deposition would breach compliance with the protocol approved by your research ethics board. If you are unable to adhere to our open data policy, please kindly revise your statement to explain your reasoning and we will seek the editor's input on an exemption. Please be assured that, once you have provided your new statement, the assessment of your exemption will not hold up the peer review process.

6. If the reviewer comments include a recommendation to cite specific previously published works, please review and evaluate these publications to determine whether they are relevant and should be cited. There is no requirement to cite these works unless the editor has indicated otherwise.

Additional Editor Comments :

After carefully reviewing both the manuscript and the reviewers’ comments, I share similar thoughts regarding the paper. I therefore recommend that the authors address all points raised by both reviewers and submit a thoroughly revised version of the manuscript.

[Note: HTML markup is below. Please do not edit.]

Reviewers' comments:

Reviewer's Responses to Questions

**Comments to the Author**

1. Is the manuscript technically sound, and do the data support the conclusions?

Reviewer #1: Partly

Reviewer #2: Yes

**********

2. Has the statistical analysis been performed appropriately and rigorously?

Reviewer #1: Yes

Reviewer #2: Yes

**********

3. Have the authors made all data underlying the findings in their manuscript fully available?

The PLOS Data policy

Reviewer #1: Yes

Reviewer #2: Yes

**********

4. Is the manuscript presented in an intelligible fashion and written in standard English?

Reviewer #1: Yes

Reviewer #2: Yes

**********

Reviewer #1: The paper presents an interesting approach that evaluated ten base models and eight ensemble configurations across two large-scale retail datasets, considering both point and

probabilistic accuracy under varying retraining frequencies. The analysis is interesting in the area of forecasting.

However, I found serious points to be noted.

* I suggest the author provide more detail on the time series. While they mention having information on products and vendors, it's unclear what type of data was used in the experiments. Was it just products per week? Please specify the size of the observation window and the size of the prediction. Also, please indicate whether it's a multi-step prediction, a single-step prediction, or a multi-variable, multi-step prediction.

* The author mentions using Nixtla. I'm a little concerned about the study's reproducibility, given that Nixtla is a paid service. How can reproducibility be guaranteed when using these platforms, which may not be accessible to everyone?

* According to the Nixtla platform used, the model training and evaluation apparently involved using prompts with LLM. It is suggested that the author provide further explanation regarding whether this training was indeed done at the prompt level, in order to validate the accuracy of the results.

*The author mentions that the parameters were selected from those used in the competitions. It would be ideal to include a table with these parameters for each model.

Furthermore, could the author clarify whether a hyperparameter search was also conducted and what strategies were used?

* The author should mention if the datasets had missing data, what percentage, and how they were addressed.

There is a some weaks:

* In section "Overview, the author should complete each section. For instance, the manuscript said:

"After a brief review of related works (Section ?), in Section we describe the design of the experiment used in our study. The datasets and their characteristics are presented in ?, while ..."

"Finally, Section ? contains our summary and conclusions."

Same situation for line 194.

* In Table 1, it is suggested to add the description of each acronym at the bottom of the table.

* Acronyms must be described first (e.g. IA, ENSACC, i.i.d).

Reviewer #2: Referee Report – PONE-D-25-56430

The cost of ensembling: is it always worth combining global retail forecasting models?

1. Summary:

This paper studies the trade-off between forecasting accuracy and computational cost in ensemble learning for global retail demand forecasting. Using the M5 and VN1 datasets, the author evaluates a range of base models and ensemble configurations under different retraining frequencies. Both point and probabilistic performance are assessed, with computational time used as a proxy for cost. The results show that ensembles generally improve accuracy, especially for probabilistic forecasts, but at a higher computational cost, particularly for accuracy-driven combinations. The paper also finds that small ensembles (2–3 models) are often sufficient, and that less frequent retraining can substantially reduce cost with little loss in accuracy.

2. Major strengths:

The paper addresses an important and timely problem, as managing the cost–accuracy trade-off is increasingly relevant in large-scale forecasting systems.

The empirical analysis is thorough and well designed. The use of two widely studied datasets, multiple base models and ensemble strategies, and different retraining scenarios provides a solid experimental setup.

The practical value of the findings is another strong point. The results offer clear guidance for practitioners, particularly regarding the benefits of small ensembles and reduced retraining frequency. The explicit link to computational cost and cloud deployment further enhances the paper’s relevance.

3. Major concerns (most of them briefly discussed by the author):

3.1. Ensemble construction: The analysis is limited to simple averaging ensembles. While this is a reasonable baseline, it restricts the scope of the conclusions. More adaptive approaches (e.g., weighted or dynamic combinations) could lead to different cost–accuracy trade-offs. At a minimum, the paper should discuss how alternative ensemble strategies might affect the results. Including an additional benchmark would strengthen the analysis.

3.2. Simplified cost modeling: The cost analysis relies on a simplified setup with fixed cloud pricing and a single hardware configuration. This does not fully reflect real-world systems, where parallelization and different pricing schemes can significantly affect costs. The author should make these assumptions explicit and discuss their implications, as the current setup may overstate the cost disadvantage of ensembles.

3.3. Statistical analysis: Although the Friedman–Nemenyi test is mentioned, the results are not reported or discussed. Most conclusions rely on descriptive comparisons, despite the fact that performance differences are often small. Stronger statistical support is needed, especially for claims about the near-optimality of small ensembles and the similarity between methods.

3.4. Data stability assumption: The analysis assumes relatively stable data, with limited discussion of concept drift or trends. This is a strong assumption in retail forecasting, where demand can change abruptly. The conclusion that less frequent retraining does not harm performance should therefore be interpreted cautiously. The authors should clarify under which conditions this result is expected to hold.

4. Minor comments: There are a few minor issues that should be addressed.

4.1. In the final paragraph of the “Related Works” section, the manuscript claims to offer both theoretical clarity and practical guidance. However, the contribution of the paper is predominantly practical in nature.

4.2. The analysis of Figure 7 in section “Results and Discussion” is confusing.

4.3. Some formatting errors need correction, e.g., missing section references.

4.4. Table 4 is quite dense and could better highlight key trade-offs.

5. Recommendation: Overall, this is a well-executed and practically relevant empirical study. The experimental design is solid, and the findings provide useful insights for both researchers and practitioners. However, the paper would benefit from clearer positioning of its contribution, stronger statistical analysis, and a more nuanced discussion of cost modeling and ensemble strategies. Addressing these points would improve both rigor and impact. I recommend minor revision.

**********

what does this mean?). If published, this will include your full peer review and any attached files.

If you choose “no”, your identity will remain anonymous but your review may still be made public.

**Do you want your identity to be public for this peer review?**  For information about this choice, including consent withdrawal, please see our Privacy Policy

Reviewer #1: No

Reviewer #2: No

**********

[NOTE: If reviewer comments were submitted as an attachment file, they will be attached to this email and accessible via the submission site. Please log into your account, locate the manuscript record, and check for the action link "View Attachments". If this link does not appear, there are no attachment files.]

To ensure your figures meet our technical requirements, please review our figure guidelines: https://journals.plos.org/plosone/s/figures

You may also use PLOS’s free figure tool, NAAS, to help you prepare publication quality figures: https://journals.plos.org/plosone/s/figures#loc-tools-for-figure-preparation.

NAAS will assess whether your figures meet our technical requirements by comparing each figure against our figure specifications.

# Author comment: Author response to Decision Letter 1

**DOI:** 10.1371/journal.pone.0336281.r002

27 May 2026

Dear Editor,

We want to thank you and the reviewers for the careful reading of our manuscript and the con-

structive feedback provided. We have carefully revised the paper to address all the comments and

suggestions.

We prepared a detailed response to each reviewer’s comment, addressing their specific requests and

clarifying our methodological choices.

Response to Editors

We sincerely thank all editors for their constructive feedback. Below, we address each comment

point by point.

• We have carefully reviewed the manuscript and supplementary files to ensure compliance with

PLOS ONE formatting and submission requirements.

• Thank you for highlighting the journal’s code sharing requirements. All author-generated code

that underpins the findings of the manuscript is already publicly available on GitHub and re-

leased under an open-source license. The repository link https://github.com/marcozanotti/global-

retrain.

• Thank you for pointing this out. We have carefully reviewed the Funding Information and

Financial Disclosure sections and corrected them so that the information is now fully consistent

across the submission.

• Thank you for the clarification. All datasets used in this study are publicly available and can

be freely shared. We have revised the Data Availability Statement accordingly to make this

explicit and to ensure full compliance with the journal’s open data policy.

Response to Reviewers

We sincerely thank all reviewers for their constructive feedback. Below, we address each comment

point by point. Reviewer comments are in italics, followed by our responses.

Reviewer 1

The paper presents an interesting approach that evaluated ten base models and eight ensemble

configurations across two large-scale retail datasets, considering both point and probabilistic accuracy

under varying retraining frequencies. The analysis is interesting in the area of forecasting.

• I suggest the author provide more detail on the time series. While they mention having informa-

tion on products and vendors, it’s unclear what type of data was used in the experiments. Was

it just products per week? Please specify the size of the observation window and the size of the

prediction. Also, please indicate whether it’s a multi-step prediction, a single-step prediction, or

a multi-variable, multi-step prediction.

We agree that additional details about the structure of the time series and the forecasting

setup improve the clarity and reproducibility of the study. In the revised manuscript, we have

expanded the description of the datasets and the forecasting task. Specifically, we clarified that

the experiments are conducted on large-scale retail datasets composed of daily (M5 dataset)

and weekly (VN1 dataset) sales time series at the product level (SKU). We now explicitly report

in a new table (Table 1): the length of the historical observation window used by the models,

the forecast horizon, and the type of forecasting task. We also clarified that the problem is

formulated as a global multi-series, multi-step forecasting task, where models are trained jointly

across many product-level time series and generate forecasts for the entire prediction horizon.

• The author mentions using Nixtla. I’m a little concerned about the study’s reproducibility, given

that Nixtla is a paid service. How can reproducibility be guaranteed when using these platforms,

which may not be accessible to everyone?

We thank the reviewer for raising this important point regarding reproducibility. We would

like to clarify that Nixtla provides both proprietary services (e.g., TimeGPT) and fully open-

source libraries. In this study, we exclusively used Nixtla’s open-source libraries, which are

freely accessible and publicly available to anyone. The models used in the experiments are

standard forecasting methods implemented in Nixtla’s open-source ecosystem (e.g., StatsFore-

cast, MLForecast, NeuralForecast). Therefore, the experimental pipeline does not rely on any

proprietary service or paid infrastructure.

• According to the Nixtla platform used, the model training and evaluation apparently involved

using prompts with LLM. It is suggested that the author provide further explanation regarding

whether this training was indeed done at the prompt level, in order to validate the accuracy of

the results.

We thank the reviewer for this comment and the opportunity to clarify a potential misunder-

standing. Nixtla provides two different types of tools: TimeGPT, a proprietary LLM-based

forecasting service that uses prompting; Open-source forecasting libraries (e.g., StatsForecast,

MLForecast, NeuralForecast), which implement classical statistical, machine learning, and deep

learning forecasting methods. In this study, we did not use TimeGPT or any LLM-based or

prompt-based methodology. All experiments were conducted using only the open-source Nixtla

libraries, which rely on conventional machine learning and deep learning models for time series

forecasting. Therefore: no prompting was used, no large language model was involved, and

model training followed standard supervised learning procedures using historical time series

data.

• The author mentions that the parameters were selected from those used in the competitions. It

would be ideal to include a table with these parameters for each model. Furthermore, could the

author clarify whether a hyperparameter search was also conducted and what strategies were

used?

We thank the reviewer for this helpful suggestion. We agree that additional clarity regard-

ing model configurations improves reproducibility and transparency. We have now included a

dedicated table reporting the main hyperparameter settings used for each model in the Sup-

plementary information. Regarding hyperparameter optimization, no additional tuning was

performed in this study. This decision was driven by computational budget constraints, as our

experimental design involves repeated retraining across multiple models and datasets, making

full hyperparameter optimization prohibitively expensive. Instead, we relied on established hy-

perparameter configurations from high-performing solutions in forecasting competitions, which

have been shown to be competitive benchmarks in similar settings.

2

• The author should mention if the datasets had missing data, what percentage, and how they

were addressed.

We thank the reviewer for raising this point. Both datasets used in this study, namely the

M5 and VN1 competition datasets, do not contain missing values. Therefore, no imputation

or additional preprocessing for missing data was required. We have clarified this point in the

revised manuscript for completeness.

• In section ”Overview, the author should complete each section. For instance, the manuscript

said: ”After a brief review of related works (Section ?), in Section we describe the design of the

experiment used in our study. The datasets and their characteristics are presented in ?, while

…” ”Finally, Section ? contains our summary and conclusions.” Same situation for line 194.

We thank the reviewer for carefully identifying these issues. We have corrected all missing

and inconsistent section references throughout the manuscript, including those in the Overview

section and line 194. The revised version now includes complete and accurate section numbering.

• In Table 1, it is suggested to add the description of each acronym at the bottom of the table.

We thank the reviewer for this suggestion. We have updated Table 1 to improve clarity and

readability.

• Acronyms must be described first (e.g. IA, ENSACC, i.i.d).

We thank the reviewer for this remark. We have revised the manuscript to ensure that all

acronyms are defined at their first occurrence in the text (e.g., IA, ENSACC, i.i.d.), as suggested.

Reviewer 2

This paper studies the trade-off between forecasting accuracy and computational cost in ensemble

learning for global retail demand forecasting. Using the M5 and VN1 datasets, the author evaluates

a range of base models and ensemble configurations under different retraining frequencies. Both

point and probabilistic performance are assessed, with computational time used as a proxy for cost.

The results show that ensembles generally improve accuracy, especially for probabilistic forecasts,

but at a higher computational cost, particularly for accuracy-driven combinations. The paper also

finds that small ensembles (2–3 models) are often sufficient, and that less frequent retraining can

substantially reduce cost with little loss in accuracy.

The paper addresses an important and timely problem, as managing the cost–accuracy trade-off is

increasingly relevant in large-scale forecasting systems. The empirical analysis is thorough and well

designed. The use of two widely studied datasets, multiple base models and ensemble strategies, and

different retraining scenarios provides a solid experimental setup. The practical value of the findings

is another strong point. The results offer clear guidance for practitioners, particularly regarding the

benefits of small ensembles and reduced retraining frequency. The explicit link to computational

cost and cloud deployment further enhances the paper’s relevance.

• Ensemble construction: The analysis is limited to simple averaging ensembles. While this is a

reasonable baseline, it restricts the scope of the conclusions. More adaptive approaches (e.g.,

weighted or dynamic combinations) could lead to different cost–accuracy trade-offs. At a min-

imum, the paper should discuss how alternative ensemble strategies might affect the results.

Including an additional benchmark would strengthen the analysis.

We thank the reviewer for this insightful suggestion. We agree that alternative ensemble

strategies such as weighted averaging, dynamically adjusted combinations, and stacking-based

approaches may potentially improve predictive accuracy by adapting model contributions or

learning optimal combination schemes. However, these methods typically introduce additional

3

computational complexity, including weight estimation procedures, validation overhead, or the

need for secondary learning models. Moreover, it is not always clear how to combine proba-

bilistic forecasts with these approaches. In this study, the focus is specifically on evaluating

the trade-off between forecasting accuracy and computational cost under repeated retraining

scenarios. For this reason, and due to computational budget constraints, we restrict our analysis

to simple averaging ensembles, which provide a strong and widely used baseline while avoiding

additional optimization or training costs. Exploring more advanced ensemble strategies was

not feasible within the computational budget available for this study, but it is pointed out as

a limitation and direction for future research. We have added a corresponding discussion in

the revised manuscript to clarify this design choice and to acknowledge that more sophisticated

ensemble methods may lead to different cost–accuracy trade-offs, albeit at higher computational

expense.

• Simplified cost modeling: The cost analysis relies on a simplified setup with fixed cloud pricing

and a single hardware configuration. This does not fully reflect real-world systems, where paral-

lelization and different pricing schemes can significantly affect costs. The author should make

these assumptions explicit and discuss their implications, as the current setup may overstate the

cost disadvantage of ensembles.

We thank the reviewer for this important observation. We agree that real-world forecasting

systems may involve a wide range of computational configurations, including different hardware

setups, pricing schemes, and levels of parallelization, all of which can influence absolute cost

estimates. However, it is not feasible to exhaustively evaluate all possible system configurations

within a single study. For this reason, we adopt a controlled experimental design with a fixed

hardware configuration and constant pricing scheme in order to ensure comparability across

models and retraining scenarios. This approach follows the design adopted in prior work on

retraining in forecasting, in particular Spiliotis E, Petropoulos F. On the update frequency of

univariate forecasting models. European Journal of Operational Research, which is, to the best

of our knowledge, the only existing study that explicitly investigates retraining frequency in

forecasting systems under a cost-aware perspective. That study also relies on a single hard-

ware configuration and fixed cost assumptions to maintain experimental consistency. We have

clarified these assumptions explicitly in the revised manuscript and added a discussion on their

implications. In particular, we acknowledge that while this setup enables a controlled com-

parison of relative computational costs, it may not capture all real-world optimizations such as

parallel execution or heterogeneous pricing environments, which could affect absolute cost levels

but are unlikely to change the qualitative conclusions regarding the cost–accuracy trade-offs.

• Statistical analysis: Although the Friedman–Nemenyi test is mentioned, the results are not re-

ported or discussed. Most conclusions rely on descriptive comparisons, despite the fact that

performance differences are often small. Stronger statistical support is needed, especially for

claims about the near-optimality of small ensembles and the similarity between methods.

We thank the reviewer for this valuable suggestion. We agree that stronger statistical support

improves the robustness of the conclusions. In response, we have now included the results of

the Friedman–Nemenyi test in the Supplementary information. These results provide a formal

statistical assessment of the performance differences among the compared methods and support

the conclusions drawn in the manuscript.

• Data stability assumption: The analysis assumes relatively stable data, with limited discussion

of concept drift or trends. This is a strong assumption in retail forecasting, where demand can

change abruptly. The conclusion that less frequent retraining does not harm performance should

therefore be interpreted cautiously. The authors should clarify under which conditions this result

4

is expected to hold.

We thank the reviewer for this important observation. We agree that retail demand forecasting

can be affected by non-stationarities and concept drift, where abrupt changes in demand pat-

terns may occur due to external events, seasonality shifts, or structural breaks. In our study, the

empirical analysis is based on two large-scale retail datasets (M5 and VN1) that already incorpo-

rate substantial heterogeneity across products, stores, and time, including intermittent demand

and varying seasonal patterns. However, we acknowledge that our experimental setup does not

explicitly isolate or model concept drift mechanisms, and therefore implicitly assumes a degree

of temporal stability over the evaluation horizon. For this reason, it is explicitly stated that

”This robustness likely stems from the relative stability of the datasets, which do not exhibit

substantial structural changes or concept drift. Under such conditions, base models continue to

capture underlying demand patterns effectively over time, and ensemble combinations further

enhance stability as retraining becomes less frequent.”. We have revised the manuscript to clar-

ify that the conclusion regarding the limited impact of reduced retraining frequency should be

interpreted under conditions of moderate and gradual data evolution, as observed in the con-

sidered datasets. In highly non-stationary environments, where abrupt structural changes or

strong regime shifts occur, more frequent retraining may still be necessary to maintain optimal

performance. This clarification has been added to the discussion section to better delineate the

scope of validity of our findings. Moreover, it is pointed out as a limitation and future research.

• In the final paragraph of the “Related Works” section, the manuscript claims to offer both the-

oretical clarity and practical guidance. However, the contribution of the paper is predominantly

practical in nature.

We thank the reviewer for this insightful observation. We agree that the main contribution of

the paper is primarily empirical and practical, focusing on experimental evidence and applied

insights. We have revised the final paragraph of the “Related Works” section to better reflect

this emphasis and to avoid overstating the theoretical contribution of the study.

• The analysis of Figure 7 in section “Results and Discussion” is confusing.

We thank the reviewer for pointing this out. The issue was due to a missing figure for the

VN1 dataset in the original version of the manuscript, which led to incomplete and potentially

confusing interpretation of Figure 7. This has now been corrected in the revised manuscript by

including the missing VN1 figure. The section is now complete.

• Some formatting errors need correction, e.g., missing section references.

We thank the reviewer for carefully identifying these issues. We have corrected all missing and

inconsistent section references throughout the manuscript.

• Table 4 is quite dense and could better highlight key trade-offs.

We thank the reviewer for this helpful suggestion. We agree that the original version of Table 4

was dense due to the simultaneous presentation of multiple metrics, model families, and ensemble

configurations. To improve readability and better highlight the key accuracy–computational cost

trade-offs, we have revised the presentation of the table. Otherwise, we will need more space to

separate the single table into many smaller ones.

We believe that these revisions substantially improve the manuscript, making its contributions

clearer, more rigorous, and better aligned with the expectations of the readership. We are grateful

for the reviewers’ insightful feedback, which has helped us to strengthen our work.

Thank you for your consideration. We look forward to your response.

Sincerely,

Attachment Submitted filename: Response to reviewers.pdf

# Peer review: Decision Letter 1

**DOI:** 10.1371/journal.pone.0336281.r003

**Authors:** Flavio Ziegelmann

10 Aug 2026

PONE-D-25-56430R1

The cost of ensembling: is it always worth combining global retail forecasting models?

PLOS One?

Dear Dr. Zanotti,

Thank you for submitting your manuscript to PLOS ONE. After careful consideration, we feel that it has merit but does not fully meet PLOS ONE’s publication criteria as it currently stands. Therefore, we invite you to submit a revised version of the manuscript that addresses the points raised during the review process.

I agree with Reviewer 1 that further improvements are needed in line with the concerns they have raised.

Please submit your revised manuscript by Sep 24 2026 11:59PM. If you will need more time than this to complete your revisions, please reply to this message or contact the journal office at plosone@plos.org. When you're ready to submit your revision, log on to https://www.editorialmanager.com/pone/ and select the 'Submissions Needing Revision' folder to locate your manuscript file.

- A letter that responds to each point raised by the academic editor and reviewer(s). You should upload this letter as a separate file labeled 'Response to Reviewers'.
- A marked-up copy of your manuscript that highlights changes made to the original version. You should upload this as a separate file labeled 'Revised Manuscript with Track Changes'.
- An unmarked version of your revised paper without tracked changes. You should upload this as a separate file labeled 'Manuscript'.

If you would like to make changes to your financial disclosure, please include your updated statement in your cover letter. Guidelines for resubmitting your figure files are available below the reviewer comments at the end of this letter.

If applicable, we recommend that you deposit your laboratory protocols in protocols.io to enhance the reproducibility of your results. Protocols.io assigns your protocol its own identifier (DOI) so that it can be cited independently in the future. For instructions see: https://journals.plos.org/plosone/s/submission-guidelines#loc-laboratory-protocols. Additionally, PLOS ONE offers an option for publishing peer-reviewed Lab Protocol articles, which describe protocols hosted on protocols.io. Read more information on sharing protocols at https://plos.org/protocols?utm_medium=editorial-email&utm_source=authorletters&utm_campaign=protocols.

As the corresponding author, your ORCID iD is verified in the submission system and will appear in the published article. PLOS supports the use of ORCID, and we encourage all coauthors to register for an ORCID iD and use it as well. Please encourage your coauthors to verify their ORCID iD within the submission system before final acceptance, as unverified ORCID iDs will not appear in the published article. *Only*  the individual author can complete the verification step; PLOS staff *cannot*  verify ORCID iDs on behalf of authors.

We look forward to receiving your revised manuscript.

Kind regards,

Flavio A. Ziegelmann, Ph.D.

Academic Editor

PLOS One

Journal Requirements:

If the reviewer comments include a recommendation to cite specific previously published works, please review and evaluate these publications to determine whether they are relevant and should be cited. There is no requirement to cite these works unless the editor has indicated otherwise.

Additional Editor Comments:

Thank you for the revised submission. After reviewing it, I agree with Reviewer 1 that further improvements are needed in line with the concerns they have raised.

[Note: HTML markup is below. Please do not edit.]

Reviewers' comments:

Reviewer's Responses to Questions

**Comments to the Author**

Reviewer #1: (No Response)

Reviewer #2: All comments have been addressed

**********

2. Is the manuscript technically sound, and do the data support the conclusions?

Reviewer #1: Yes

Reviewer #2: Yes

**********

3. Has the statistical analysis been performed appropriately and rigorously?

Reviewer #1: Yes

Reviewer #2: Yes

**********

4. Have the authors made all data underlying the findings in their manuscript fully available?

The PLOS Data policy

Reviewer #1: Yes

Reviewer #2: Yes

**********

5. Is the manuscript presented in an intelligible fashion and written in standard English?

Reviewer #1: Yes

Reviewer #2: Yes

**********

Reviewer #1: Dear author, thank you for addressing the previous comments. I consider the current version to be clear and concise. However, I have a couple of additional comments.

* It is important to mention that the link to the GitHub repository does not exist. Could you please check it?

* The author states: "Given a historical observation window of length n, the model produces forecasts for a fixed horizon h, generating all future values recursively." Table 1 subsequently lists the values n = 4 years and h = 28.

I am still wondering: is the model input a single variable? Does this variable span a 4-year period? What is the size of the time step—1 hour, 1 day, 1 week, or 1 month?

Furthermore, is the model output a multi-step prediction? That is, are 28 steps generated for a single variable? Would these steps represent hours, days, or months?

I understand that M5 has a horizon of 28 and VN1 has one of 13; But please, could you clarify what the output looks like? Specifically, the time step?

I gather that the model input covers 4 years (though the time step size isn't clear to me yet) and that the output has a size of 28 (again, the time step size isn't clear). Also, I'd like to know if we are dealing with a single variable or multiple variables.

Along these same lines, would it be possible for the author to provide a graph showing the input steps and the output steps? This would greatly help in classifying the generated model.

* If I may, one additional suggestion is to include a diagram showing the complete pipeline; this would be ideal for understanding the applied methodology.

Reviewer #2: (No Response)

**********

what does this mean?). If published, this will include your full peer review and any attached files.

If you choose “no”, your identity will remain anonymous but your review may still be made public.

**Do you want your identity to be public for this peer review?**  For information about this choice, including consent withdrawal, please see our Privacy Policy

Reviewer #1: No

Reviewer #2: No

**********

[NOTE: If reviewer comments were submitted as an attachment file, they will be attached to this email and accessible via the submission site. Please log into your account, locate the manuscript record, and check for the action link "View Attachments". If this link does not appear, there are no attachment files.]

To ensure your figures meet our technical requirements, please review our figure guidelines: https://journals.plos.org/plosone/s/figures

You may also use PLOS’s free figure tool, NAAS, to help you prepare publication quality figures: https://journals.plos.org/plosone/s/figures#loc-tools-for-figure-preparation.

NAAS will assess whether your figures meet our technical requirements by comparing each figure against our figure specifications.

# Author comment: Author response to Decision Letter 2

**DOI:** 10.1371/journal.pone.0336281.r004

12 Aug 2026

We thank the Reviewer for pointing this out. Re-reading the sentence, we agree it conflates three distinct quantities — the length of the training sample, the length of the input window used to build model inputs, and the forecast horizon — and that Table 1 did not state the time step unambiguously. We answer each question in turn and have revised the manuscript accordingly.

\textbf{Time step}. The time step is one day for M5 and one week for VN1. The values in parentheses in Table 1 (7 and 52) were the seasonal periods, not the granularity; we have separated these into two rows to remove the ambiguity.

\textbf{Single or multiple variables}. The target is a single variable: unit sales (demand). Each time series records the units sold of one product at one location per time step — SKU × store for M5 (30,490 series) and product × warehouse for VN1 (15,053 series). We therefore forecast one variable, not a vector of jointly -modelled interdependent variables.

The model inputs, however, are multivariate. For each series and each forecast origin the input vector comprises: (i) lagged values of the target; (ii) rolling and expanding statistics of the target; (iii) calendar variables (day-of-week, week, month, year); (iv) static categorical identifiers (store, item, category, department, state for M5; retailer, warehouse, product for VN1); and (v) for M5, exogenous event and price/promotion variables. The setting is thus univariate forecasting with covariates, estimated globally.

\textbf{What "global" means here}. A single model is estimated jointly on all series of a dataset — the parameters are shared across series, and the training sample is the pooled panel of all series (Eq 1). We do not fit one model per series.

\textbf{The 4 or 3 years is the training sample, not the input window}. The value n in the corrected Table 1 (1,460 daily observations for M5; 157 weekly for VN1) is the length of the initial training sample available per series, from which the model is estimated. It is not the input passed to the model at prediction time. Because we use an expanding-window rolling-origin evaluation, the input window grows as the origin advances by the step size, which we set equal to 1.

\textbf{Output}. The output is a multi-step-ahead path for the same single target variable: h consecutive future values of unit sales, at the same granularity as the input. For M5, h = 28 corresponds to the next 28 days (four weeks); for VN1, h = 13 corresponds to the next 13 weeks (approximately one quarter). Both are business-relevant replenishment horizons in retail.

Concretely, for M5 at origin t and series i, the model returns the predicted units sold of product i at its store on days t+1, …, t+28. With a step size of one, origins advance one day at a time across the one-year test set, yielding 336 origins per series and almost 10.3M forecast paths in total; all reported metrics are averaged over origins and series.

The newly added Figure 1 helps understand the whole process.

\textbf{Recursive versus direct}. We are grateful for the opportunity to correct an imprecision: the strategy is not recursive for all models. The machine learning models (mlforecast) use a recursive strategy — a one-step-ahead model applied iteratively, with predictions fed back as lags. The deep learning models (neuralforecast) use a direct multi-output strategy, emitting all h values in a single forward pass. The original sentence over-generalised the former to all methods; the revised text distinguishes them explicitly.

We deliberately retained each framework's native strategy rather than forcing a common one, since our objective is to compare methods as they are actually deployed, and imposing a non-native strategy would have required re-implementing several architectures and would have distorted the computing-time comparison that is central to the study.

\item \textit{If I may, one additional suggestion is to include a diagram showing the complete pipeline; this would be ideal for understanding the applied methodology.}

We thank the Reviewer for this suggestion. We have added (in the supplementary material) a new figure presenting the complete experimental pipeline, from data preparation through model estimation, rolling-origin evaluation under the retraining scenarios, post-hoc ensemble construction, and the accuracy, probabilistic, and cost evaluations. Together with Fig 1, we believe this makes the applied methodology substantially easier to follow.

Attachment Submitted filename: response02.pdf

# Peer review: Decision Letter 2

**DOI:** 10.1371/journal.pone.0336281.r005

**Authors:** Flavio Ziegelmann

15 Sep 2026

The cost of ensembling: is it always worth combining global retail forecasting models?

PONE-D-25-56430R2

Dear Dr. Zanotti,

We’re pleased to inform you that your manuscript has been judged scientifically suitable for publication and will be formally accepted for publication once it meets all outstanding technical requirements.

Within one week, you’ll receive an e-mail detailing the required amendments. When these have been addressed, you’ll receive a formal acceptance letter and your manuscript will be scheduled for publication.

An invoice will be generated when your article is formally accepted. Please note, if your institution has a publishing partnership with PLOS and your article meets the relevant criteria, all or part of your publication costs will be covered. Please make sure your user information is up-to-date by logging into Editorial Manager at Editorial Manager® and clicking the ‘Update My Information' link at the top of the page. For questions related to billing, please contact billing support.

If your institution or institutions have a press office, please notify them about your upcoming paper to help maximize its impact. If they’ll be preparing press materials, please inform our press team as soon as possible -- no later than 48 hours after receiving the formal acceptance. Your manuscript will remain under strict press embargo until 2 pm Eastern Time on the date of publication. For more information, please contact onepress@plos.org.

Kind regards,

Flavio A. Ziegelmann, Ph.D.

Academic Editor

PLOS One

Additional Editor Comments (optional):

Reviewers' comments:

Reviewer's Responses to Questions

**Comments to the Author**

Reviewer #1: All comments have been addressed

**********

2. Is the manuscript technically sound, and do the data support the conclusions?

Reviewer #1: Yes

**********

3. Has the statistical analysis been performed appropriately and rigorously?

Reviewer #1: Yes

**********

4. Have the authors made all data underlying the findings in their manuscript fully available?

The PLOS Data policy

Reviewer #1: Yes

**********

5. Is the manuscript presented in an intelligible fashion and written in standard English?

Reviewer #1: Yes

**********

Reviewer #1: Dear authors, thank you for addressing the comments. I consider the current version to be

clear and concise. I have no further comments.

**********

what does this mean?). If published, this will include your full peer review and any attached files.

If you choose “no”, your identity will remain anonymous but your review may still be made public.

**Do you want your identity to be public for this peer review?**  For information about this choice, including consent withdrawal, please see our Privacy Policy

Reviewer #1: No

**********

# Editor report: Acceptance letter

**DOI:** 10.1371/journal.pone.0336281.r006

**Authors:** Flavio Ziegelmann

PONE-D-25-56430R2

PLOS One

Dear Dr. Zanotti,

I'm pleased to inform you that your manuscript has been deemed suitable for publication in PLOS One. Congratulations! Your manuscript is now being handed over to our production team.

At this stage, our production department will prepare your paper for publication. This includes ensuring the following:

* All references, tables, and figures are properly cited

* All relevant supporting information is included in the manuscript submission,

* There are no issues that prevent the paper from being properly typeset

You will receive further instructions from the production team, including instructions on how to review your proof when it is ready. Please keep in mind that we are working through a large volume of accepted articles, so please give us a few days to review your paper and let you know the next and final steps.

Lastly, if your institution or institutions have a press office, please let them know about your upcoming paper now to help maximize its impact. If they'll be preparing press materials, please inform our press team within the next 48 hours. Your manuscript will remain under strict press embargo until 2 pm Eastern Time on the date of publication. For more information, please contact onepress@plos.org.

You will receive an invoice from PLOS for your publication fee after your manuscript has reached the completed accept phase. If you receive an email requesting payment before acceptance or for any other service, this may be a phishing scheme. Learn how to identify phishing emails and protect your accounts at https://explore.plos.org/phishing.

If we can help with anything else, please email us at customercare@plos.org.

Thank you for submitting your work to PLOS One and supporting open access.

Kind regards,

PLOS One Editorial Office Staff

on behalf of

Professor Flavio A. Ziegelmann

Academic Editor

PLOS One
